# Proteome‐Wide Mendelian Randomization Identifies Candidate Causal Proteins for Cardiovascular Diseases

**DOI:** 10.1002/ggn2.202500003

**Published:** 2025-03-10

**Authors:** Chen Li, Nicolas De Jay, Shan‐Shan Zhang, Xin Fang, Supriya Sharma, Katrina A. Catalano, Venkatesh Sridharan, Zhaoqing Wang, Lei Zhao, Joseph D. Szustakowski, Ching‐Pin Chang, Joseph C. Maranville, Emily R. Holzinger, Erika M. Kvikstad

**Affiliations:** ^1^ Informatics and Predictive Sciences Bristol‐Myers Squibb Cambridge 02141 USA; ^2^ Immunology and Cardiovascular Thematic Research Center Bristol‐Myers Squibb Cambridge Brisbane 94005 USA; ^3^ Translational Medicine Bristol‐Myers Squibb Cambridge Lawrenceville 08540 USA

**Keywords:** cardiovascular diseases, Mendelian randomization, multi‐omics, proteome‐wide, single cell

## Abstract

Integration of human genomics and other omics across different ancestries provides novel, affordable, and systematic approach for target identification. We used Mendelian randomization approaches to unravel causal associations between 2,940 circulating proteins and 19 CVD. We found 218 proteins that impacted risk of one or more CVDs through forward MR (106 and 182 using cis‐pQTLs only and cis‐ + trans‐pQTLs, respectively), among which 107 were previously reported as associated with CVD or CVD‐related traits. There were 102 proteins replicated (FDR < 5%, 53 with cis‐pQTLs only and 88 with cis‐ + trans‐pQTLs) using the FinnGen Olink data. BTN3A2 was highlighted as a novel candidate gene for ischemic stroke, suggesting a crosstalk between immune modulation and stroke pathogenesis. Single cell integration prioritized PAM for stable angina pectoris and ventricular arrhythmia and LPL for peripheral artery disease, whose transcriptional expressions were enriched in cardiomyocytes. Forward and reverse MR found largely non‐overlapping proteins (only 2 overlapped: LGALS4 and MMP12), suggesting distinct proteomic causes and consequences of CVD. Our study provides human genetics‐based evidence of novel candidate genes, a foundational step towards full‐scale causal human biology‐based drug discovery for CVD.

## Graphical abstract

Here, causal relations are examined between 2940 plasma proteins and 19 cardiovascular diseases (CVD) using a human genetics‐based method. 218 proteins are found whose blood levels influence CVD risks, with about half being new discoveries and largely confirmed in an independent cohort. Some of these proteins are present in heart muscle cells, highlighting potential new targets for heart diseases.

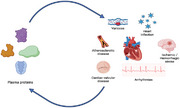

## 1 Introduction

Recent development in novel therapeutic treatments for cardiovascular disease (CVD) has helped to improve survival and reduce hospitalization of patients.^[^ ^1^, ^2^ ^]^ Despite this progress, CVD remains the leading cause of death across ancestries and geographies.^[^ ^3^ ^]^ Innovation and investments in developing new therapies for CVD have successfully driven discovery of many novel drugs, including some first‐ and best‐in‐class therapies, such as mavacamten, a targeted inhibitor of cardiac myosin, for treatment of patients with obstructive hypertrophic cardiomyopathy;^[^ ^4^ ^]^ inhibitors of SGLT2 to treat heart failure regardless of left ventricle ejection fraction and diabetes comorbidity;^[^ ^5^, ^6^, ^7^ ^]^ vericiguat, a stimulator of sGC, to treat heart failure patients with reduced ejection fraction;^[^ ^8^ ^]^ inhibitors of PCSK9 for hypercholesterolemia and atherosclerotic CVD.^[^ ^9^, ^10^ ^]^ However, in recent years, the number of drugs entering all phases of clinical trials and drugs approved by regulatory bodies (including the US Food and Drug Administration) for CVD has declined, especially when comparing to drugs in other therapeutic areas, such as oncology.^[^ ^11^ ^]^ This is possibly due to a relatively higher cost of trials, as cardiovascular trials are often larger in size and longer in duration to manifest primary endpoints that satisfy regulatory requirements.^[^ ^12^ ^]^ The challenges confronted in cardiovascular drug development call for new approaches to increase accuracy and efficiency of trials at lower costs, and studies have shown drug targets with robust human genetic evidence are more likely to be successful in clinical trials.^[^ ^13^ ^]^

Recent advances in omics technologies provide opportunities to discover novel therapeutic strategies in an unbiased, rapid, and cost‐effective manner. High throughput immunoaffinity‐based proteomics platform is emergingly applied to systematically quantify protein abundance in large cohorts,^[^ ^14^ ^]^ and 97% of therapeutic targets for FDA‐approved drugs as of 2016 are proteins.^[^ ^15^ ^]^ Deep profiling of circulating proteins with integration of genomics in a large population cohort allows us to understand proteins associated with disease at an unprecedented scale with the possibility of unraveling novel pathways involved in CVD pathogenesis. Observational studies have shown cross‐sectional associations between proteins and certain CVD,^[^ ^16^ ^]^ but causal relationships are unclear due to potential confounding effects and reverse causations. Genome‐wide association studies (GWAS) of circulating proteins identify genetic variants in cells that regulate protein expression that in turn may have effects on protein levels in plasma, and these genetic variants can be used as instrumental variables (IVs) to infer causality of protein expression on CVD risk under the framework of Mendelian randomization (MR).

Studies have demonstrated the feasibility of MR methodology in identifying protein targets for CVD but are often restricted to a small number of targeted proteins, one or a few CVD and one ancestry population.^[^ ^17^, ^18^, ^19^ ^]^ In this study, we leveraged the largest genetic mapping of circulating proteome that has recently been published as a public data resource, which measures 2940 proteins in up to 50 000 individuals in UK Biobank,^[^ ^20^ ^]^ to systematically identify disease associations across 19 CVD ranging from rare conditions of myocarditis and cardiomyopathy to common conditions of heart failure and ischemic stroke. To distinguish potential targets whose protein expression levels affect disease risks and biomarkers that vary protein expressions as consequences of diseases, we conducted forward and reverse MR to delineate bi‐directional causality. We prioritized newly identified targets and biomarkers with further evidence from cross‐database evaluation of protein pathophysiological function and clinical implication. We also analyzed potential therapeutic and adverse effects on a wide spectrum of diseases to respectively show opportunities for drug repurposing and caveats for safety issues.

## 2 Experimental Section

### 2.1 Study Overview

**Figure 1:**
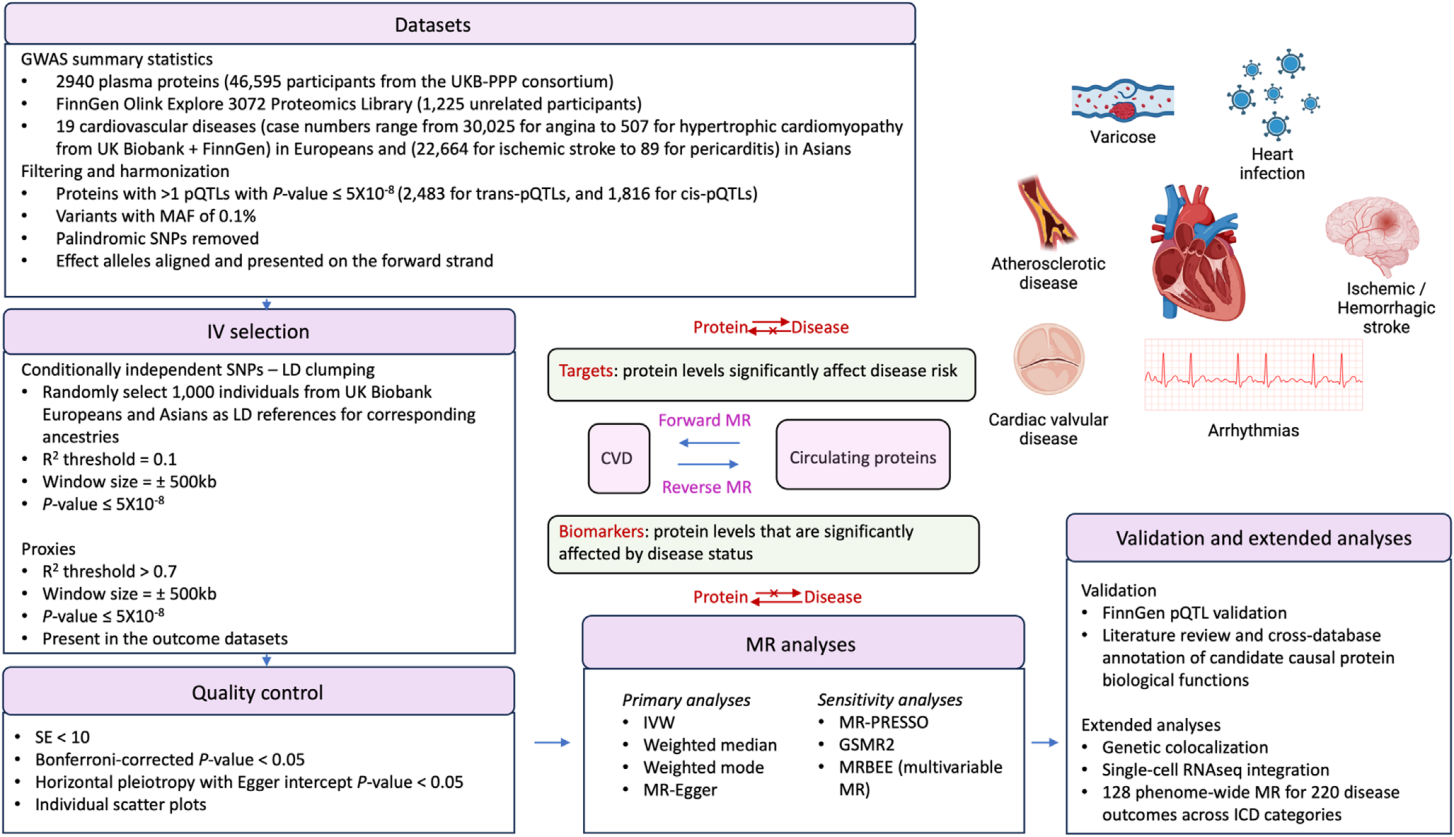
Workflow of the causal inference between plasma proteins and CVD. GWAS: genome‐wide association study, pQTL: protein quantitative trait loci, EUR: European, ASI: Asian, LD: linkage disequilibrium, CVD: cardiovascular disease, IVs: instrumental variables, MR: Mendelian randomization, SE: standard error, IVW: inverse variance‐weighted. Created with BioRender.com.

The overview of study design – data processing and analysis workflow was summarized in the **Figure** **1**. The study consisted of two parts, the primary analysis, a bi‐directional MR that disentangled causal relationships between proteins and CVD, and secondary analyses to strengthen evidence for target prioritization, including single cell integration to assess cell‐type enrichment of targets prioritized and differential gene expression between cardiomyopathy and healthy cardiac cell types; manual curation of biological functions and cross‐database annotation of protein druggability and disease association; a phenome‐wide MR scan to assess pleiotropy and specificity of targets and biomarkers across disease categories.

### 2.2 MR Analyses

Various methods have been developed to conduct MR with different capabilities in type I and type II error control, and degrees of tolerance to invalid instruments.^[^ ^21^ ^]^ We used the inverse variance weighted MR as the primary method because of its conceptually simple design and relatively greater power to detect less significant but true positive results.^[^ ^22^ ^]^ Other MR methods used included MR Egger,^[^ ^23^ ^]^ median weighted^24^ ^]^ and mode weighted MR.^[^ ^25^ ^]^ In the forward MR sensitivity analyses, we used MR‐PRESSO^26^ ^]^ and GSMR2^[^ ^27^, ^28^ ^]^ to detect and adjust for horizontal pleiotropy and MRBEE^29^ ^]^ for a multivariable MR analysis that takes account of shared associations of *trans‐*pQTLs with multiple proteins.

#### 2.2.1 Forward‐MR

Forward MR investigated causal roles of proteins on CVD risk, where proteins were exposures and CVD were outcomes. IVs for proteins were derived from summary statistics from the largest GWAS of plasma proteins to date, where genetic associations were assessed in 2940 plasma proteins in 46 595 UK Biobank participants, of which 34 557 participants were of European ancestry, 931 participants were African, 920 participants were of Central/South Asia, 308 participants were from Middle East, 262 participants were East Asians and 97 participants were admixed Americans.^[^ ^20^ ^]^ We assessed European and East Asian cohorts because of data availability on the CVD outcomes. Independent lead variants (also known as protein quantitative trait loci, or pQTLs) with *P*‐value < 5 × 10^−8^ were selected as IVs for European and Asian cohorts separately (Linkage disequilibrium (LD) clumped variants – variants in LD R^2^ < 0.1 with each other and located within 500 kb around lead variants). LD was calculated based on a randomly selected subset of Caucasian (n = 10 000) or all Asian (n = 2783) participants in the UK Biobank for Europeans and Asians, respectively.^[^ ^30^ ^]^ For IVs absent from the CVD GWAS datasets, we found the best proxies available (*P*‐value < 5 × 10^−8^, in LD R^2^ > 0.7 with index variants, and within 500 kb distance from the index variants). We assessed *cis‐*pQTLs only and *cis‐* + *trans‐*pQTLs as IVs separately. *cis‐*pQTLs were defined as genetic variants within 1Mb on either side of the start and the stop codon of a protein‐coding gene. Proteins that significantly affected CVD risk with Bonferroni‐adjusted *P*‐value < 0.05 (adjusted for the total number of proteins and diseases tested) were identified as target candidates and followed‐up with downstream analyses of cross‐database protein annotation, single cell enrichment analyses and phenome‐wide MR scan.

Candidate causal proteins were validated using pQTLs from FinnGen Olink analyses. These pQTLs were derived from 1225 unrelated Finnish samples measured with Olink Explore 3072 library. The same MR methods were used as in the discovery analyses, with *cis‐*pQTLs only and *cis‐* + *trans‐*pQTLs as IVs, separately.

We conducted sensitivity analyses to adjust for horizontal pleiotropy for all significant associations using *cis‐* + *trans‐*pQTLs as IVs. In these analyses, we removed highly pleiotropic variants through comparing observed and expected residual sum of squares (outlier significance threshold = Bonferroni‐corrected *P*‐value < 0.05 for the number of IVs used for each pair of associations) using the MR‐PRESSO or the HEIDI‐outlier tests (HEIDI < 0.01) using the GSMR2. Additionally, to account for *trans‐*pQTLs shared between proteins, we conducted a multivariable MR analysis using MRBEE that estimated an independent effect on each CVD for significant proteins identified with *cis‐* + *trans‐*pQTLs as IVs. In this analysis, we used z‐scores from joint tests for independent variants (LD‐clumped with R^2^ < 0.01 and a 500 kb window) associated with at least one protein.

#### 2.2.2 Reverse‐MR

Reverse MR studied protein level changes caused by CVD, where diseases were exposures and proteins were outcomes. IVs for CVD were independent lead variants (LD clumped variants with *P*‐value < 5 × 10^−8^, in LD R^2^ < 0.1 between each other and located within 500 kb around lead variants) derived from summary statistics of GWAS on 19 CVD where CVD were identified by ICD‐10 and phecode in Europeans (UK Biobank and FinnGen meta‐analysis, n = 449 000) and Asians (Biobank Japan, n = 179 000). Sample size for each CVD was listed in Table S1 (Supporting Information). Disease definition was described in detail in Sakaue S et al.^[^ ^31^ ^]^ Briefly, disease phenotypes were curated from text‐mining of electronic medical records in the Biobank Japan and mapped to phecode and ICD10 in the FinnGen and UK Biobank. Full GWAS summary statistics were downloaded from Sakaue S et al.^[^ ^31^ ^]^ IV definition for each CVD was developed based on this GWAS meta‐analysis for Europeans and Asians separately. For hypertrophic and dilated cardiomyopathy, IVs were built from a more cardiomyopathy‐focused GWAS with greater statistical power for Europeans.^[^ ^32^ ^]^ Ancestry‐specific LD was estimated in corresponding cohorts as illustrated in the Forward‐MR analysis. For IVs absent from the proteomics GWAS datasets, we found the best proxies available using the same criteria as described in the forward‐MR analysis. Proteins that were significantly affected by CVD (Bonferroni‐adjusted *P‐*value < 0.05) were identified as potential biomarkers and compared against significant results from the forward‐MR to rule out reverse causation.

### 2.3 Cross‐Database Protein Annotation

Protein biological functions were annotated with cross‐referencing multiple databases, including UniProt and RefSeq. We also annotated protein druggability by interrogating Drug‐Gene‐Interaction (DGI) database,^[^ ^33^ ^]^ ChEMBL v33,^[^ ^34^ ^]^ PharmGKB,^[^ ^35^ ^]^ and DrugCentral Postgres v14.5^36^ ^]^ databases to find therapeutic targets that has been approved or under clinical development. To evaluate protein associations with diseases, we queried GWAS Catalog^37^ ^]^ for population‐level evidence, as well as ClinVar^38^ ^]^ and ClinGen^39^ ^]^ for clinically reported implications.

### 2.4 Phenome‐Wide MR Scan

Significant proteins from the forward‐ and reverse‐MR primary analyses were followed up with the phenome‐wide MR scan to detect opportunities for drug repurposing and potential safety concerns. There were 128 disease outcomes with ≥50 cases included in the analysis, covering a wide range of categories, with an average of 8 diseases representing each category. The analysis was performed in a bi‐directional manner in European and Asian cohorts separately. For Asian cohort, an additional 38 quantitative biomarkers and 23 medication usage phenotypes were tested. Diseases were defined by ICD‐10 and phecode by a previous GWAS meta‐analysis in UK Biobank, FinnGen and Biobank Japan, where detailed description of the 3 cohorts and disease definitions were shown.^[^ ^31^ ^]^ The diseases covered a wide range of categories, with an average of 8 diseases representing each category. Enrichment was assessed via the over representation analysis to determine whether a protein functional group (G) was over‐represented in a disease category (D), and enrichment *P*‐value was calculated under hypergeometric distribution using the formula: P=1−∑i=0k−1MiN−Mn−iNn, where N is the product of the total number of diseases across disease categories and the total number of proteins in G, M is the product of the total number of diseases in one disease category D and the total number of proteins in G, n is the number of significant protein signals in G across disease categories, and k is the number of significant protein signals in G for one disease category D. Enrichment significance threshold was Bonferroni‐corrected for the total number of protein functional groups and the total number of disease categories.

### 2.5 Genetic Colocalization Analysis

For each significant pair of associations identified in the forward MR using cis‐pQTLs, we assessed the probability that genetic associations were shared between the protein, the CVD and corresponding gene expression levels across tissues using HyPrColoc Bayesian divisive clustering algorithm.^[^ ^40^ ^]^ Default settings of parameters were used, including a prior probability of 1 × 10^−4^, a vector of conditional colocalization priors of 0.005, 0.01, and 0.02, and regional and alignment probability thresholds of 0.6 to 0.9 with an increment of 0.1.

### 2.6 Single‐Cell Analysis

To link circulating proteins to proteins enriched in cell types originating in the heart tissues and demonstrate specificity for cardiomyopathy versus healthy hearts, we analyzed cell type enrichment for significant genetic signals from MR analyses using single‐cell RNA sequencing data from left ventricular samples and assessed differential gene expression between cardiomyopathy and healthy hearts.

#### 2.6.1 Single‐Cell RNA Sequencing Data Curation

We curated publicly available single‐cell RNA sequencing data from a recently published cohort of 42 human left ventricular samples (11 DCM, 15 HCM, 16 non‐failing hearts).^[^ ^41^ ^]^ Cell and cell‐type metadata, and gene counts adjusted for ambient RNA were retrieved from the Broad Institute's Single Cell Portal (accession SCP1303). Cell‐type enrichment and differential gene expression data were retrieved from figures and supplementary tables of the paper by Chaffin M et al.^[^ ^41^ ^]^

#### 2.6.2 Cell‐Type Enrichment

To determine whether a gene exhibited significantly enriched expression in one or more cell types, pseudobulk profiles were generated from healthy and patient donors.^[^ ^41^ ^]^ A gene was considered to be enriched for a cell type if it satisfied the following criteria: (1) the gene expression was >4X higher for the cell type compared to the other cell types (one versus the other) with FDR_BH_‐adjusted *P*‐value < 0.01, (2) at least 25% of nuclei in the cell type expressed the gene, and (3) a classifier trained on the gene expression predicted whether a nucleus belonged to the cell type with AUC >0.6. For visualization, we included genes if their expressions were significantly enriched in at least one cell type and sorted them according to (1) the cell type in which nuclei displayed the highest average expression, (2) the average Gini coefficient calculated from the average nuclei expression and the percentage of nuclei expressing that gene.

#### 2.6.3 Differential Gene Expression Between Cardiomyopathy and Healthy Hearts

Pseudobulk profiles were analyzed using a limma‐voom model regressing gene expression in each cell type on a disease group (HCM versus control and DCM versus control) adjusted for age and sex.^[^ ^41^ ^]^ A gene was differentially expressed if the expression was 50% higher in the cardiomyopathy than non‐failing cardiac cell types with a FDR_BH_‐adjusted *P*‐value < 0.01.

#### 2.6.4 Gene Expression Visualization

For visualization, gene counts were summed into pseudobulk profiles by donor and cell type and filtered as described above. Filtered gene counts were normalized using the trimmed mean of M‐values method using *edgeR::calcNormFactors(method = “TMM”)* to account for library size differences between pseudobulk profiles and standardized into a common scale using log_2_ counts per million (CPM) with *edgeR::cpm(log = T, pseudocount = 0.25)*.

### 2.7 Analysis Software

All analyses were performed using the statistical software R (Version 4.1.3). We used the PLINK 1.9 beta and 2.0 alpha (http://pngu.mgh.harvard.edu/purcell/plink/) to generate LD matrices and perform LD clumping;^[^ ^46^ ^]^ the TwoSampleMR (https://github.com/MRCIEU/TwoSampleMR),^[^ ^42^ ^]^ MR‐PRESSO (https://github.com/rondolab/MR‐PRESSO),^[^ ^26^ ^]^ GSMR2 (https://github.com/jianyanglab/gsmr2)^[^ ^27^, ^28^ ^]^ and MRBEE (https://github.com/noahlorinczcomi/MRBEE)^29^ ^]^ R packages to perform MR analyses, the HyPrColoc R package (https://github.com/cnfoley/hyprcoloc) to perform multi‐trait colocalization,^[^ ^40^ ^]^ and the Seurat (https://satijalab.org/seurat/) and the edgeR R packages to perform single cell analyses.^[^ ^43^, ^44^ ^]^

### 2.8 Ethical Statement

The MR analyses were based on publicly available GWAS summary statistics and ancestry‐specific LD matrices were estimated using UK Biobank individual‐level data (Applications 26 041 and 65 851). The included GWAS all received informed consent from study participants and have been approved by pertinent local institutional review board.

## 3 Results

### 3.1 Target Discovery with Forward‐MR – Causal Effects of Proteins on CVD Risks

Forward‐MR analysis identified 218 proteins that significantly affected at least one CVD risk, among which 106 proteins were identified using *cis‐*pQTLs only (*P*‐value < 1.74 × 10^−7^) and 182 proteins using *cis‐ + trans‐*pQTLs as IVs (*P*‐value < 1.27 × 10^−7^) in the European ancestry, which spanned multiple Olink panels, including cardiometabolic, inflammation, neurology, and oncology panels (Figure S1 and Table S5, Supporting Information). Among these causal protein candidates, 107 were previously reported to be associated with CVD or CVD‐related traits, including therapeutic drug targets that have been approved or with ongoing clinical trials, such as PCSK9 for myocardial infarction, stroke, and coronary revascularization, ANGPTL3 for reducing low‐density lipoprotein cholesterol, and ECE1 for congestive heart failure and hypertension (Table S2, Supporting Information). Many of the targets (67.4%) identified were supported by strong literature evidence for a role in immune response and atherosclerotic lesion formation, vascular remodeling, myogenesis and energy metabolism (Table S3, Supporting Information). Most of the significant associations remained robust in sensitivity analyses correcting for horizontal pleiotropy (86% and 90% of significant pairs identified using cis‐ + trans‐pQTLs reached Bonferroni‐adjusted *P*‐value threshold after outlier filtering using MR‐PRESSO and GSMR methods, respectively, Tables S12 and S13, Supporting Information). In a multivariable MR, 44 proteins showed likely independent causal effects on at least one CVD (*P*‐value < 0.05, Table S14, Supporting Information), including LPA for angina and cardiac valvular disease.

**Figure 2:**
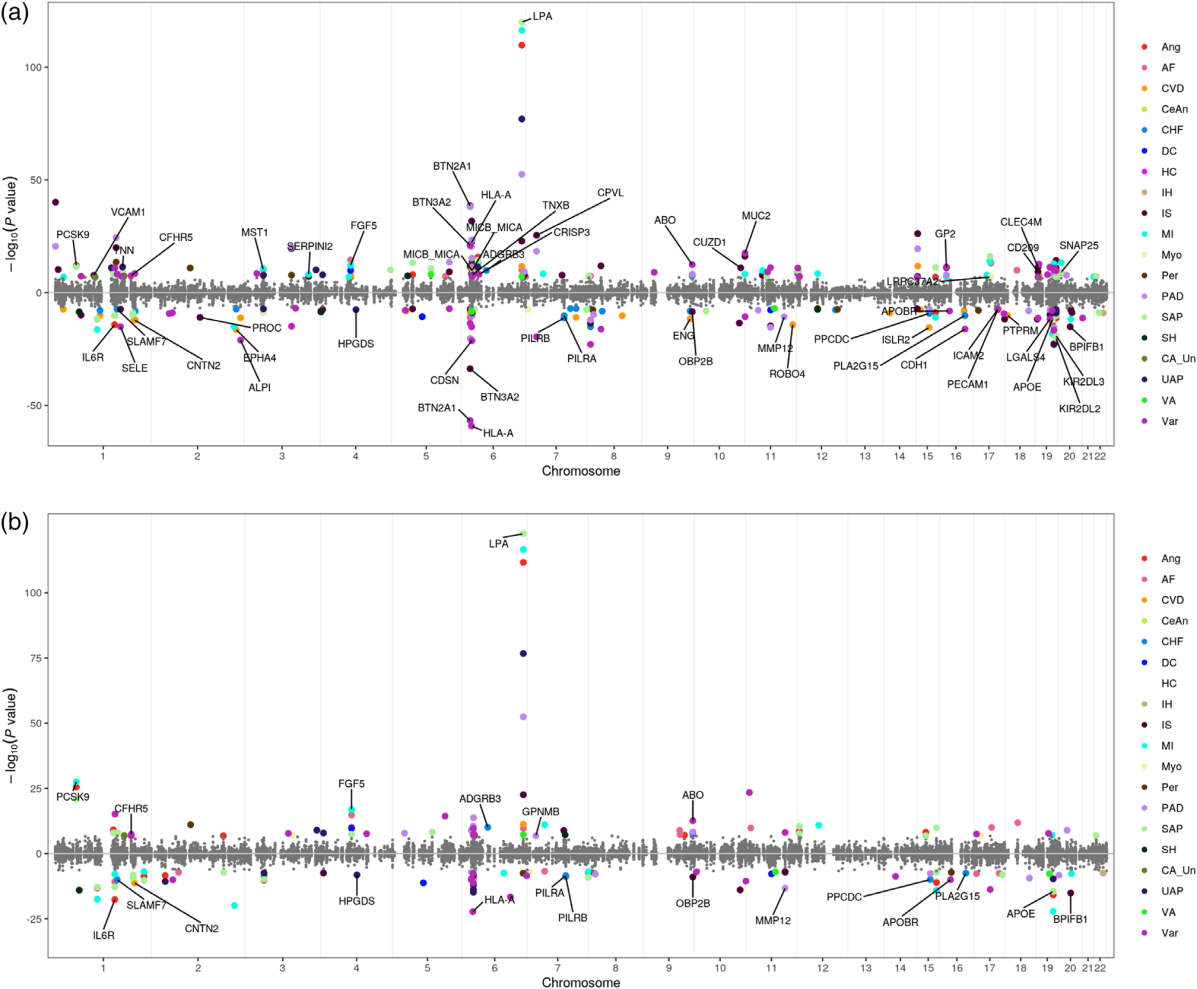
Discovery of targets. Causal protein‐to‐disease associations are shown. The Miami plots demonstrate forward‐MR results using A. cis‐ and trans‐pQTLs or B. cis‐pQTLs only as instrumental variables and inverse variance weighted method in the European ancestry. Proteins labeled with their protein names are those replicated in the FinnGen cohort (Bonferroni‐adjusted *P*‐value < 0.05). Y‐axis shows ‐log_10_ (*P*‐value), and X‐axis shows the genomic coordinates of start positions of gene coding sequences. Each dot represents a pair of protein‐disease association. Significant pairs are colored with each color representing one cardiovascular disease. Associations with positive and negative beta coefficients are split into two parts with a horizontal line at y = 0. The upper part shows associations with positive beta coefficients, indicating an antagonism mechanism of targeting, and the lower part shows associations with negative beta coefficients, indicating an agonism mechanism. Ang: Angina pectoris, AF: Atrial flutter/fibrillation, CVD: Cardiac valvular disease, CeAn: Cerebral aneurysm, CHF: Chronic heart failure, DC: Dilated cardiomyopathy, HC: Hypertrophic cardiomyopathy, IH: Intracerebral hemorrhage, IS: Ischemic stroke, MI: Myocardial infarction, Myo: Myocarditis, Per: Pericarditis, PAD: Peripheral arterial disease, SAP: Stable angina pectoris, SH: Subarachnoid hemorrhage, CA_Un: Unruptured cerebral aneurysm, UAP: Unstable angina pectoris, Var: Varicose veins, VA: Ventricular arrhythmia.

There was a total of 125 out of 218 (57.3%) proteins replicated (*P*‐value < 0.05, 68 with cis‐pQTLs only and 106 with cis‐ + trans‐pQTLs) from the FinnGen Olink analyses, 102 proteins reached FDR_BH_‐adjusted *P*‐value < 0.05 (53 with *cis‐*pQTLs only and 88 with *cis‐ + trans‐*pQTLs), and 57 proteins (21 with *cis‐*pQTLs only and 56 with *cis‐ + trans‐*pQTLs) reached Bonferroni corrected significance threshold (**Figure** **2**; Figure S2, Supporting Information). Most of the validated targets also showed consistent directionality of effects (Table S11, Supporting Information).

**Figure 3:**
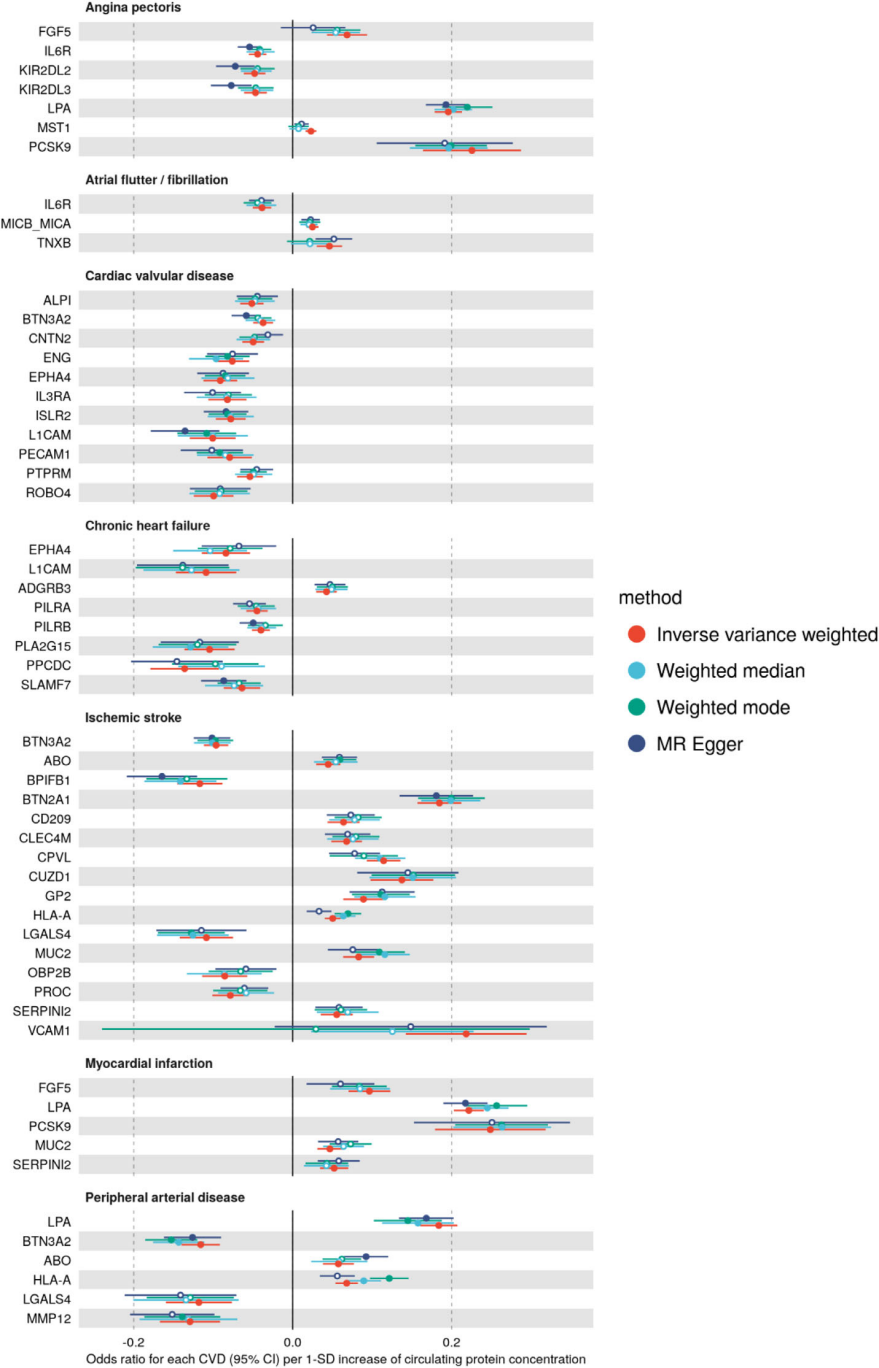
Causal protein effects on CVD that are replicated in FinnGen. Protein effects on CVD are presented as odds ratios (95% CI) per 1‐SD increment of genetically predicted circulating protein concentration. Colors indicate 4 different MR methods. Significant results after Bonferroni correction are displayed as filled circles, while non‐significant results as hollow circles. All protein‐to‐disease associations replicated using the FinnGen proteomics data are shown in the Figure S2. This plot shows a subset of CVD that are largest in case numbers.

Among novel targets, BTN3A2 had a relatively large protective effect on ischemic stroke (**Figure** **3**; Figure S3, Supporting Information), and the results were consistent across 4 MR methods with balanced horizontal pleiotropy tested with MR Egger intercept (Table S10, Supporting Information). The causal association remained significant when using only *cis‐*pQTLs as IVs (Table S5, Supporting Information). BTN3A2 belongs to the butyrophilin family that has recently been shown to be involved in Vγ9Vδ2 T cell activation– a main subtype of circulating T cells with the γδ receptor, thereby initiating immune responses against tumor cells and pathogens.^[^ ^45^, ^46^ ^]^ Novel drugs activating butyrophilin 3A proteins for solid tumors and hematological malignancies are under investigation.^[^ ^47^ ^]^ BTN3A2 exhibited upregulated mRNA expression in myocardial tissues of hypertrophic cardiomyopathy patients.^[^ ^48^ ^]^ Shared genetic associations have been identified via genetic colocalization analyses for circulating protein and transcriptional gene expression levels of BTN3A2 in heart left ventricle and various anatomical regions of the brain.^[^ ^20^ ^]^ A recent MR study found genetically predicted BTN3A2 transcriptional expression level in the brain was positively associated with lacunar stroke risk and other cardiac imaging markers of cerebral small vessel disease.^[^ ^49^ ^]^ Loss‐of‐function variants of the BTN3A2 gene were associated with hypertension and a genome‐wide association study identified BTN3A2 as a candidate causal gene for left ventricular mass.^[^ ^50^, ^51^ ^]^ Here we showed higher BTN3A2 concentrations in the blood decreased risks for ischemic stroke, suggesting a role of immune modulation in ischemic stroke pathophysiology and targeting butyrophilin family proteins may hold promises as a therapeutic strategy for ischemic stroke. Moreover, BTN2A1 is another protein in the butyrophilin protein family and it binds to CD209 on monocytes and dendritic cells, through which mediates internalization of various pathogens.^[^ ^52^ ^]^ Genetic associations with plasma protein levels of BTN2A1 were colocalized with its gene expression levels in the heart left ventricles.^[^ ^20^ ^]^ We found BTN2A1 and CD209 were on our validated list of causal candidate proteins for ischemic stroke (Figure 3), recapitulating the potential involvement of BTN‐mediated immune responses in pathogenesis of ischemic stroke.

Around 45% significant proteins affected only one CVD, while several proteins were causally associated with a highly pleiotropic set of diseases across CVD (Figure 2, Figure S4 and Table S5, Supporting Information), such as LPA with 18 different CVD, including stable and unstable angina pectoris, myocardial infarction and peripheral arterial disease, possibly through dual pathological attributes of lipoprotein(a) in procoagulant effects of apo(a) and atherogenic and proinflammatory effects of oxidized apoB‐related phospholipids.^[^ ^53^ ^]^ Studies suggested LPA as an emerging therapeutic target for reducing lipoprotein(a) levels, thereby lowering risks of atherosclerotic CVD independent of lowering low‐density lipoprotein cholesterol levels,^[^ ^54^ ^]^ with positive results reported by several ongoing clinical trials.^[^ ^55^ ^]^

### 3.2 Biomarker Discovery with Reverse‐MR – Causal Effects of CVD on Protein Concentrations

**Figure 4:**
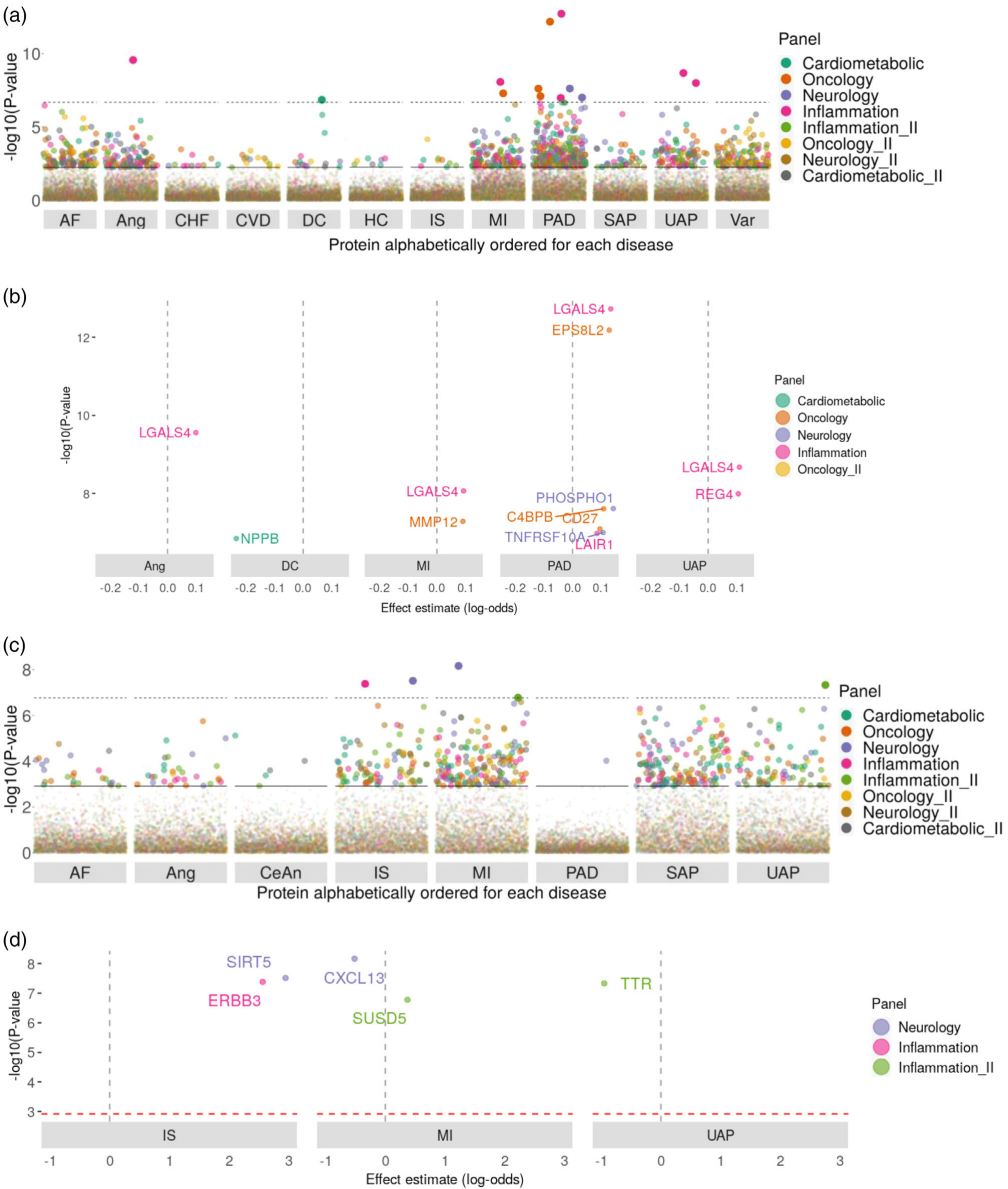
Discovery of biomarkers – circulating proteins whose concentrations are affected by CVD status. These plots show the reverse‐MR results in two ancestries using inverse variance weighted method. Proteome‐wide Manhattan plot – causal effect of each CVD on each protein in the A) (European) and C) (Asian) ancestries. CV diseases that have at least one instrumental variable are shown. Dashed line indicates Bonferroni‐corrected *P*‐value threshold and solid line indicates FDR < 0.05. Significant causal associations are shown in the panels B) (European) and D) (Asian). Effect coefficient β is plotted as X‐axis and ‐log_10_(*P* ‐value) as Y‐axis. Dashed vertical line indicates β = 0. AF: Atrial flutter/fibrillation, Ang: Angina pectoris, CHF: Chronic heart failure, CVD: Cardiac valvular disease, CeAn: Cerebral aneurysm, DC: Dilated cardiomyopathy, HC: Hypertrophic cardiomyopathy, IS: Ischemic stroke, MI: Myocardial infarction, PAD: Peripheral arterial disease, SAP: Supplemental Table angina pectoris, UAP: Unstable angina pectoris, Var: Varicose veins.

We identified 15 biomarkers, among which 5 were from the analyses in the Asian population (ERBB3, SIRT5, CXCL13, SUSD5, TTR, Tables S4 and S6, Supporting Information; **Figure** **4**). Four of the 11 biomarkers identified in the European population (MMP12, LGALS4, C4BPB, and LAIR1) were replicated using FinnGen Olink proteomics data (P‐value < 0.05), and C4BPB reached FDR_BH_‐adjusted *P*‐value < 0.05 significance threshold (Table S11, Supporting Information). Of note, ERBB3, a member of the membrane‐bound tyrosine kinase receptor family that activates cell proliferation and differentiation, found only in the Asian population, has recently been shown to have cardioprotective effects on cardiomyocyte survival and angiogenesis under stress (Table S4, Supporting Information).^[^ ^56^ ^]^ Genetic polymorphisms of ERBB3 were reported to be associated with coronary artery disease in the Han and Uygur ancestries of China,^[^ ^57^ ^]^ and circulating protein levels of ERBB3 were associated with overweight‐related hypertension in the same population.^[^ ^58^ ^]^ Only 2 biomarkers (LGALS4, MMP12) were overlapped with the targets, suggesting distinct proteomic causes and consequences of CVD.

We also found NPPB, the gene encoding preprohormone (propro‐B‐type natriuretic peptide, preproBNP) in cardiomyocytes, as a biomarker specifically to dilated cardiomyopathy. The preproBNP is cleaved into proBNP, and then further processed into 2 circulating fragments – the biologically active BNP and the inactive N‐terminal proBNP, both of which are routinely used in clinical diagnosis and treatment management in heart failure. Studies have suggested that BNP may play different roles in heart failure with preserved and reduced ejection fraction (HFpEF and HFrEF, respectively),^[^ ^59^ ^]^ and BNP or NT‐proBNP were less effective biomarkers for HFpEF due to their average lower values in HFpEF than in HFrEF, which can drop into a normal range in some patients.^[^ ^60^ ^]^ Our finding is consistent with these studies that BNP‐related biomarkers were mainly indicative for heart diseases with reduced left ventricle ejection fraction.

### 3.3 Phenome‐Wide MR Screening for Specificity and Pleiotropy of Targets and Biomarkers

**Figure 5:**
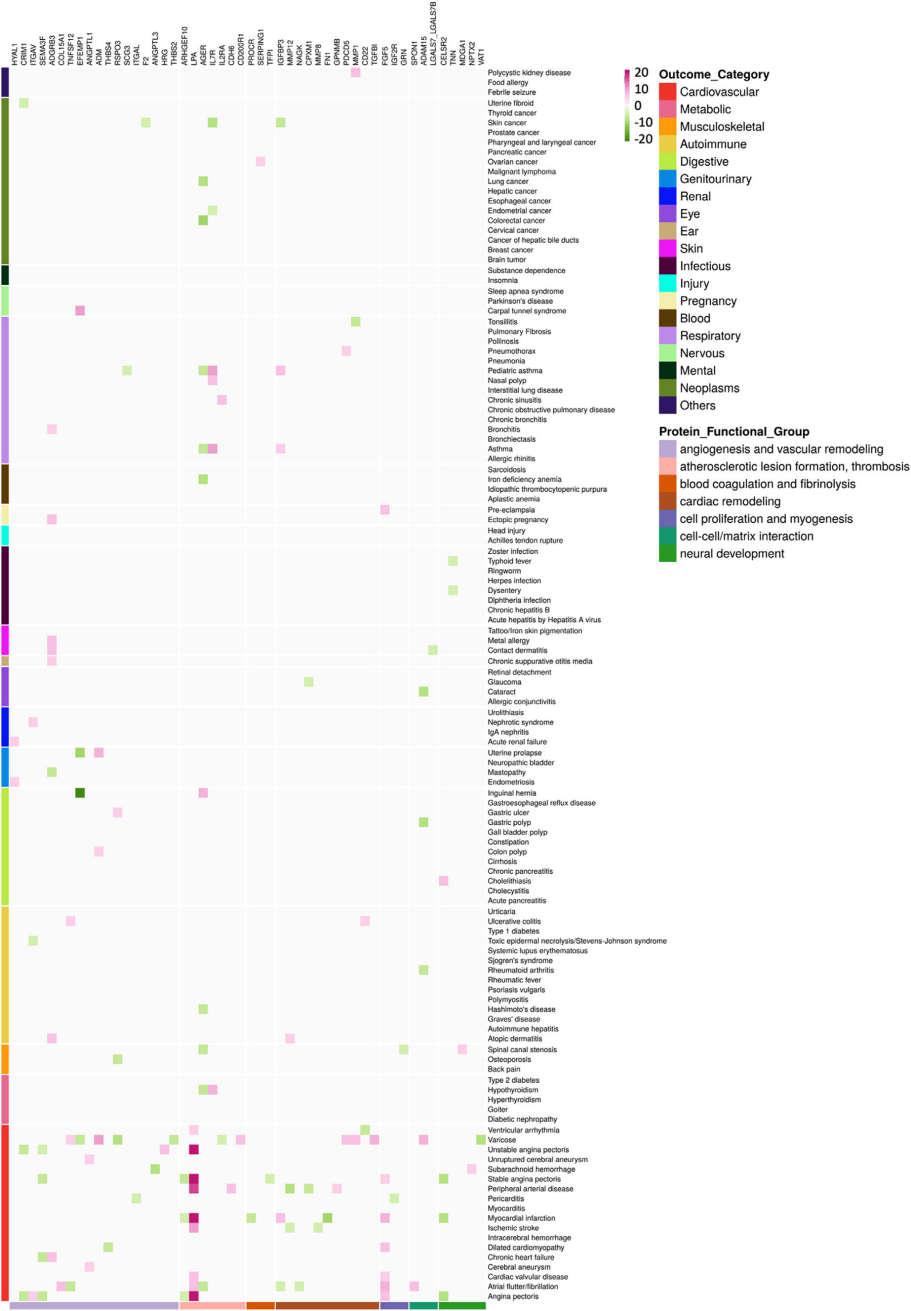
Phenome‐wide MR scan to evaluate target and biomarker specificity and pleiotropy. Z‐scores from forward‐MR results using cis‐pQTLs as instrumental variables are shown in the heatmap. For Z‐scores of absolute values >20, they are truncated to a maximum absolute value of 20. The color gradient, from green (negative) to red (positive), illustrates direction and magnitude of causal associations between proteins (X‐axis) and diseases (Y‐axis). Diseases and proteins are grouped by their different categories and labeled with different colors on the left and the bottom of the heatmap, respectively. Full phenome‐wide MR scan results are shown in the Figure S4 (Supporting Information). A subset of those results is shown here to illustrate the significantly enriched protein functional groups for their causality on CVD, including neural development, cell‐cell/matrix interaction, cardiac remodeling, atherosclerotic lesion formation and thrombosis, blood coagulation and fibrinolysis, angiogenesis and vascular remodeling as well as cell proliferation and myogenesis.

We expanded our analysis to evaluate causal relationships between significant proteins and 128 diseases across disease categories in the forward and reverse MR in the two ancestries to assess pleiotropy and specificity of targets and biomarkers that trigger or respond to one or multiple diseases (Table S7, Supporting Information). For targets, 32 out of 218 targets were specific to one disease and 58 to one disease category; for biomarkers, 3 out of 15 biomarkers were specific to one disease (Figure S4, Supporting Information). Several functional groups showed significant enrichment for their causality on CVD and not the other disease categories, including blood coagulation and fibrinolysis (enrichment *P*‐value = 2.06 × 10^−8^), angiogenesis and vascular remodeling (7.82 × 10^−11^), and cardiac remodeling (1.77 × 10^−7^, **Figure** **5**; Table S8, Supporting Information).

To assess pleiotropy, 41% (16 out of 39) proteins in the functional group of immune and inflammatory response exerted significant causal effects on at least 20 different diseases across disease categories, whereas in the other top 10 largest functional groups, there were ≤2 proteins implicated with ≥20 diseases (Table S9, Supporting Information). The pleiotropic signature of the immune and inflammatory response group was consistent when altering the pleiotropy threshold to 15, 10, or 5 proteins, suggesting involvement of this functional group in the pathogenesis of a variety of diseases across disease categories. Apart from this group, several other proteins of different functional groups showed extensive pleiotropy, such as CTSB and MST1 in regulating autophagy and apoptosis, and TNXB in mediating cell‐cell and cell‐matrix interaction (Figure S4, Supporting Information). Biomarkers were associated with a smaller number of diseases on average than targets (mean [SD]: 4.20 [2.86] versus 10.04 [14.67]), and they were not enriched for any disease categories.

### 3.4 Integration of Bulk and Single Cell Transcriptional Gene Expression Elucidates Tissue‐Specific and Cell Context‐Dependent Mechanisms of Candidate Causal Proteins in CVD Pathogenesis

To investigate mechanisms of candidate causal proteins at a tissue‐level, we analyzed genetic colocalization for 1) each pair of plasma protein levels and CVD (protein‐CVD), 2) each pair of transcriptional gene expression levels across 49 tissues and CVD (RNA‐CVD), and 3) each triad of plasma protein, tissue‐specific gene expression and CVD risk (protein‐RNA‐CVD) for significant candidate causal proteins identified with cis‐pQTLs (Table S15, Supporting Information). Thirty‐two pairs of CVD and plasma protein reached posterior probability of 0.8, indicating over 80% probability of shared genetic architecture between a CVD and a protein level measured in plasma. In addition, 26 pairs of CVD and gene expression levels in at least one tissue and 14 triads of CVD‐RNA‐protein also reached posterior probability of 0.8. While most of genes modulated CVD risk through gene expression changes in various tissues, some exerted effects specifically in just one tissue, for example, FGF5 modified disease risks for angina, myocardial infarction and atrial fibrillation all in the kidney cortex, and PROCR gene expression in the heart atrial appendage tissue may change risk of myocardial infarction.

**Figure 6:**
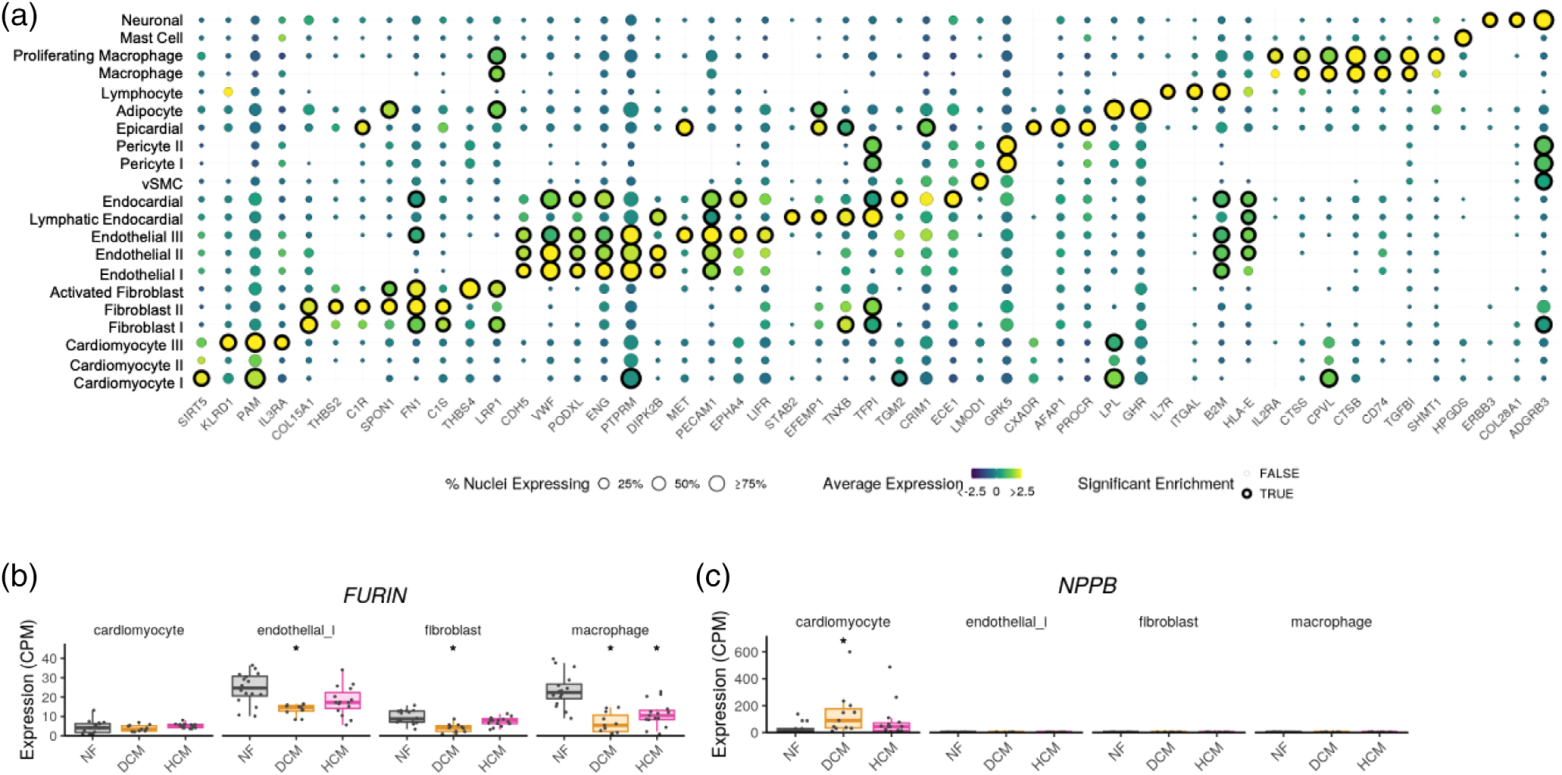
Cardiac Cell Type‐Specific Gene Expression in Cardiomyopathy. A) Overall enrichment of transcriptional expression of genes in 42 left ventricle biopsies from 11 dilated cardiomyopathy (DCM), 15 hypertrophic cardiomyopathy (HCM) and 16 non‐failing (NF) hearts combined^[^ ^41^ ^]^ Proteins with significantly enriched gene expression in at least one cell type are shown. Average expression is scaled to center at 0 and capped to a range of ‐2.5 to 2.5 with a color gradient from yellow to purple. The percentage of nuclei expressing each gene is proportional to the size of the dots. Dots are omitted for genes with less than 0.5% of nuclei within a cell type. Significant cell type enrichment is highlighted with black circles around the dots. B‐C) Box plots illustrating expression of *FURIN* and *NPPB* in non‐failing, DCM and HCM heart samples, stratified by cell types. Asterisks highlight genes with significant differential expression relative to NF.

To gain further insights into possible cell context‐dependent pathological mechanisms in the heart, we examined gene expression patterns of candidate causal proteins in major cardiac cell types of the left ventricle samples based on a recently published single‐cell late‐stage DCM and HCM heart atlas.^[^ ^41^ ^]^ Fifty‐one out of 236 unique corresponding genes (21.6%) had significantly enriched gene expressions in at least one major cardiac cell population (**Figure** **6**A; Table S16, Supporting Information). The enriched cell types of each protein were in line with its biological functions. For example, *PAM*, a known marker of cardiomyocytes encoding the major atrial membrane protein involved in proANP containing secretory granule biosynthesis, was enriched in cardiomyocytes. Similarly, *LPL*, which encodes a key enzyme in hydrolysis of triglyceride and catabolism of triglyceride‐rich lipoprotein, was significantly elevated in cardiomyocytes and adipocytes (Figure S5A, Supporting Information). Mislocalization of LPL to the cell surface of cardiomyocytes has been associated with cardiomyopathy pathogenesis in mice.^[^ ^61^ ^]^ Our findings suggest that cell‐type‐dependent mechanisms of targets emerge as compelling focal points for potential therapeutic hypotheses development.

Next, we investigated whether our prioritized targets were differentially expressed in cardiomyopathy and healthy donor heart biopsies in a cell‐type specific manner. Indeed, 95 proteins (40.2%) were differentially expressed at the transcriptional level in at least one cardiac cell type (Figure S5B and Table S17, Supporting Information). *NPPB* showed significantly higher expression in cardiomyocytes from DCM, but not HCM, than non‐failing hearts (Figure 6B). FURIN, a protein that mediates proteolytic cleavage of non‐functional proBNP to its active hormone BNP, expressed at a significantly lower level in fibroblasts, endothelial cells, and macrophages from DCM than non‐failing hearts (Figure 6C), suggesting agonists of FURIN may help restore cardioprotective activity of BNP in DCM.

## 4 Discussion

### 4.1 Main Findings

Leveraging genetic IVs to infer causalities between circulating proteins and incident diseases in a proteome‐wide and phenome‐wide manner helps to identify candidate drivers of diseases and biomarkers at an unprecedently large scale. In this study, we integrated proteomics MR with single cell analysis to prioritize novel targets for CVD. We found 218 causal signals, the majority of which conforms to known disease biology through pathways such as immune response and atherosclerotic lesion formation, vascular remodeling, myogenesis and energy metabolism. Reverse‐MR identified 15 biomarkers whose protein expressions were affected by disease status, 5 of which were exclusively found in the Asian population, demonstrating the value of multi‐ancestry populations in identifying drug targets via genetic studies of molecular traits (omics). Only two of the targets were also biomarkers, the LGALS4 and the MMP12, suggesting distinct causes and consequences of circulating proteins on CVD. Some established targets for cardiovascular drugs approved or in clinical development have been found, such as PCSK9 inhibitors (bococizumab, alirocumab and evolocumab), ECE1 inhibitor (daglutril) and ANGPTL3 inhibitor (evinacumab), supporting the value of our approach in identifying actionable druggable therapeutic targets. About half of the candidate causal genes (107/218) have been linked to CVD or CVD‐related risk factors in previous GWAS, genetic linkage or clinical studies, and the rest of genes were implicated with CVD for the first time. Many proteins (125/218) were replicated using FinnGen independent proteomics dataset. Among the novel candidate causal genes to highlight, BTN3A2 showed a relatively large effect on ischemic stroke, and the significant result was consistent across different datasets and methods, suggesting a crosstalk between immune modulation and stroke pathogenesis. Single cell integration further prioritized PAM for stable angina pectoris and ventricular arrythmia and LPL for peripheral artery disease, whose transcriptional expressions were enriched in cardiomyocytes. Our findings provide evidence supporting therapeutic hypotheses underpinning established or ongoing cardiovascular drug development and more broadly showcase the potential of large‐scale integration of multi‐omics in understanding causal human biology of complex diseases for novel drug target identification.

### 4.2 BTN3A2 as a Potential Novel Therapeutic Target for CVD

We identified candidate causal proteins for CVDs, particularly through modulating immune response and atherosclerotic lesion formation, vascular remodeling, myogenesis and energy metabolism. Among these potential novel therapeutic targets, BTN3A2 stands out as a promising target for ischemic stroke. Based on the MR results and literature review, we proposed that higher BTN3A2 levels may decrease the risk of ischemic stroke by dampening detrimental immune reactions that may exacerbate ischemic injury. BTN3A2 belongs to the butyrophilin family, which is involved in immune modulation, particularly through the activation of Vγ9Vδ2 T cells. These cells are a subset of γδ T cells that play a protective role in inflammation by recognizing stress‐induced molecules upregulated in immune responses to tumor cells and infections.^[^ ^62^ ^]^ Agonists of BTN3A2 can activate the Vγ9Vδ2 T cells that provide immune surveillance and reduce neuroinflammation and tissue damage in ischemic stroke. Previous immune‐related therapeutic approaches for stroke focus on inhibiting pro‐inflammatory cytokines, such as IL‐6 or TNF‐α, which are involved in the inflammatory response and neuroinflammation post‐stroke. For example, IL‐6 has been widely studied for its role in stroke, and therapies aimed at blocking IL‐6 have shown promise in reducing infarct size and improving recovery of ischemic stroke patients in randomized controlled trials and systematic review of prospective studies.^[^ ^63^, ^64^ ^]^ Similarly, TNF‐α inhibitors have been explored as potential stroke therapies due to their ability to reduce neuroinflammation and brain tissue damage.^[^ ^65^ ^]^ BTN3A2 shares some similarities with the existing stroke‐related immune targets, in which they are all involved in attenuating post‐stroke inflammation, but BTN3A2 offers a distinct mechanism of action, in which agonists of BTN3A2 reset and restore homeostasis of immune system through activation of the Vγ9Vδ2 T cells, rather than broadly suppressing immune responses seen with cytokine inhibitors.^[^ ^66^ ^]^ The specialized subset of T cells – Vγ9Vδ2 T cells function as sentinels to guard and repair inflammation‐induced neuronal cell stress and damage that occur in ischemic stroke. In addition, BTN3A2's potential to regulate immune responses through its interaction with other butyrophilins, such as BTN2A1, which is involved in pathogen recognition and immune activation, further supports the hypothesis that butyrophilins‐mediated Vγ9Vδ2 T cell activation can modulate stroke‐related immune mechanisms in a more precise way compared to traditional anti‐inflammatory therapies. This may represent a novel therapeutic avenue for ischemic stroke that balances immune regulation and tissue protection rather than suppressing the immune system globally.

To advance the potential novel therapies targeting BTN3A2, integrating the MR findings with functional validation is essential. The next step in validating this target involves using approaches such as CRISPR‐based gene editing and genetically modified mouse models to test the biological effects of these proteins in vitro and in vivo.

### 4.3 The Values and Limitations of Bidirectionality in MR Analyses

Observational studies are unable to distinguish causation from association due to confounding and difficulties in ascertaining directionality. We applied a bi‐directional MR approach to disentangle causal roles of circulating proteins on disease risks – to discover targets, and causal effects of diseases on circulating proteins – to identify biomarkers. Testing reverse causation allows us to distinguish causes from consequences of CVD at a proteome‐wide level. Previous MR studies that have systematically examined influences of plasma proteome on complex diseases also used the bi‐directional MR approach to distinguish candidate causal proteins from reverse causality and showed few overlapping signals between forward and reverse causality.^[^ ^67^ ^]^ Our study also found mostly non‐overlapping signals from bi‐directional associations between CVD and plasma protein levels, albeit with larger sample sizes and expanded panels of proteins measured, suggesting the causes and consequences of CVD differ at a protein level. There are limitations of the reverse MR, for example, there may be shared genetic variants associated with disease exposures and protein outcomes, which may violate the exclusion restriction assumption of the MR, where the IV must not be associated with the outcome, except through the exposure; the genetic instruments for the disease exposures may influence the protein outcomes through pathways other than the specific diseases of interest, therefore violating the assumption of exchangeability. Moreover, there are challenges in translating the findings from reverse MR into concrete clinical conclusions, because the biomarkers identified in the reverse MR simply reflect a consequence of a disease in plasma protein levels and may not necessarily be predictive of a disease risk.

One of the key questions to further pursue to fully understand protein‐level changes in different stages during disease development is to distinguish disease incidence and progression, and thereby to assess proteomic changes underpinning disease progression, but we are unable to address this due to limitations on disease outcome definitions from genome‐wide meta‐analyses. To achieve the full potential of bi‐directional MR and characterize dynamic influence of proteomic changes along disease trajectories, a longitudinal GWAS on progression endpoints will be needed. MMP12 is a notable example, of which the mechanism of action can be further elucidated with the longitudinal data. It was reported that MMP12 was upregulated after myocardial infarction, and then attenuated by endogenous inhibitors, such as tissue inhibitor of metalloproteinases (TIMPs) that provides a negative feedback loop to regulate a temporal succession of events that promote myocardial wound healing while limiting tissue damage.^[^ ^68^ ^]^ MMP12 levels are temporally fine‐tuned after myocardial infarction, orchestrating a series of events including inflammation, fibrosis, angiogenesis and collagen degradation in order to achieve optimal scar formation and prevent left ventricular dysfunction and heart failure prognosis.^[^ ^69^ ^]^ In our findings, we confirmed that MMP12 was a biomarker that increased expression after myocardial infarction, and activation of MMP12 reduced risk of peripheral arterial disease and ischemic stroke, but because of limitations on data availability of the exact time point when MMP12 protein level was quantified after ischemic injury, we were not able to assess the temporal regulation of MMP12 during cardiac remodeling upon injury. Further studies that include disease progression endpoints can help corroborate and extend our findings to characterize dynamic protein level changes of cardiovascular targets and biomarkers.

### 4.4 Pleiotropy in Proteomic MR

There are two levels of pleiotropy in proteomic MR analyses. One is at the variant level, in which pQTLs are associated with more than one protein; the other is at the protein level, where proteins can be causally associated with more than one disease.

For pleiotropy at the variant level, both *cis*‐pQTLs and *trans*‐pQTLs can be associated with multiple proteins, with *cis*‐pQTLs being physically proximal to sentinel variants, and therefore may be less likely to violate the MR assumption of pleiotropy.^[^ ^70^, ^71^, ^72^ ^]^ However, there are values of including *trans*‐pQTLs in the proteomic MR analysis, because *trans*‐pQTLs include more IVs that provide greater statistical power and the genes annotated with *trans*‐pQTLs may have biological roles in regulating homeostasis of the associated proteins through signaling transduction pathways or protein‐protein interaction.^[^ ^67^ ^]^ In the forward‐MR, we identified 112 (51.2%) targets uniquely found in the *cis‐ + trans‐*pQTLs‐based forward MR analysis, accounting for 47.6% of targets with pathophysiological roles in CVD, suggesting the value of using *trans*‐pQTLs in the MR analysis for exploring novel therapeutic hypotheses. To account for pleiotropic effects of trans‐pQTLs, we performed sensitivity analyses that can correct for pleiotropic effects, and most of the significant associations remained robust after filtering pleiotropic outliers using MR‐PRESSO and GSMR methods. We also performed a multivariable MR, where multiple proteins with shared genetic instruments were analyzed as exposures and independent effects of individual proteins were estimated. The multivariable MR causal framework can be particularly useful for proteomic MR analyses, because it can adjust for proteins that may be involved in other biological mechanisms, when genetic instruments – pQTLs are associated with multiple proteins. There may still be residual pleiotropy not adjusted for even after various sensitivity analyses having been performed, and MR is only one approach to provide causal human biology evidence‐based target nomination, other orthogonal methods are needed to fully evaluate the targets, such as systematic literature review, functional follow‐up experiments and so on.

For pleiotropy at the protein level, we identified several proteins with significant pleiotropic effects across multiple CVDs. Notably, LPA influenced a range of CVDs, including myocardial infarction, angina, atrial fibrillation and peripheral arterial disease. The causal effects of LPA on all these CVDs are in the same direction – higher protein levels of LPA lead to higher risk of CVDs, suggesting the antagonism mechanism. This suggests that lowering LPA levels can potentially yield therapeutic benefits across multiple cardiovascular conditions that are diverse in pathological mechanisms. However, there are also proteins that are associated with multiple diseases across disease categories, which are enriched in the immune response and inflammation, and their directionality of effects is not necessarily the same. Targeting such proteins may introduce unintended side effects due to their involvement in distinct pathways that are relevant to different disease manifestations. For instance, while the inhibition of proteins involved in inflammatory pathways may hold promise for CVD, the same proteins may be implicated in other diseases like cancer or autoimmune disorders, where modulation could either enhance or mitigate therapeutic outcomes. Characterizing the pleiotropic landscape uncovered by MR can inform more targeted, effective, and safer therapeutic strategies for CVD for both the design of new drugs and the evaluation of existing therapies.

### 4.5 Benefits and Limitations in Incorporating Multi‐Ancestry Element in MR Study

Leveraging multi‐ancestry data for drug discovery can help discover novel candidate causal genes. For example, two nonsense mutations of PCSK9 first reported in humans were found in an African‐American population and associated with a substantial reduction of low‐density lipoprotein cholesterol.^[^ ^73^ ^]^ This finding demonstrated that a lifelong inhibition of PCSK9 protected against coronary heart disease without noticeable safety issues. The multi‐ancestry element has recently been increasingly incorporated in population genetic studies, such as GWAS, fine‐mapping and polygenic risk score studies, but was rarely included in MR studies.^[^ ^74^, ^75^ ^]^ In our study, we identified 5 biomarkers exclusively detected in the Asian population, highlighting the values of leveraging multi‐ancestorial populations in identifying candidate causal signals. We benefited from a relatively large sample size of the Biobank Japan, one of the largest non‐European population cohorts with genome‐wide genetic and electronic medical records data available, which provided adequate statistical power in constructing IVs of disease endpoints and thereby detecting significant signals in the reverse MR analysis. We were limited by a relatively small sample size of the pQTL study in the Asian population, hindering us from identifying more potential targets in the forward MR analysis.

### 4.6 Advantage and Limitation in Integrating Bulk and Single‐Cell Transcriptome with MR Study

Candidate causal proteins identified by the MR analyses were circulating proteins, while pathological changes in CVD often occur in heart and other peripheral tissues. Although the plasma proteome is partially composed of tissue‐derived proteins, dynamics of plasma proteome alone cannot fully represent tissue‐specific changes of protein profiles.^[^ ^76^ ^]^ The plasma protein profiles are more likely to be a result of cumulative effects of protein expression changes in different tissues, for example, proteins with enriched expression in cardiomyocytes and normally absent from plasma are detected in the plasma, which can possibly be due to cardiac cell necrosis and leakage of intracellular components in pathological conditions.^[^ ^77^ ^]^ Integration of the MR signals with differentially expressed genes identified by bulk and single‐cell transcriptome analysis allows us to begin to uncover genetic mechanisms of CVD at tissue and cardiac cellular levels. There are some limitations to be pointed out, for example, compared to the plasma proteomic data described here, bulk tissue and single‐cell transcriptomic samples are smaller and more susceptible to study heterogeneity. They also cover a limited range of cardiovascular indications due to the logistical, technical, and ethical challenges in obtaining and processing fresh tissues.

### 4.7 Impact of Applying Different Methods for Multiple Testing Correction

We noted that Bonferroni‐correction that corrected for the total number of proteins and phenotypes analyzed in the phenome‐wide MR scan can be overly stringent due to intercorrelation within proteins and phenotypes. Using the Bonferroni approach controlled type 1 error with a slight loss in power, given the relatively low success rates for investigational targets passing through clinical trial development,^[^ ^78^ ^]^ the strict control on false positives at the first step of target discovery can help prioritize targets for downstream development. If using a more lenient approach to adjust for multiple testing, we may find a larger number of significant proteins, but more of which can be false positives that require extensive functional assessments with labor‐intensive and time‐consuming experiments developed on animal models. External validation using independent cohorts can validate our results on top of a stringent significance threshold applied, and we used FinnGen proteomics data to validate the results.

### 4.8 Other Limitations

There is a sample overlap between genetic associations with the proteins and most CVD we analyzed, as the former were estimated from a subset of UK Biobank cohort and the latter from a meta‐analysis of UK Biobank and FinnGen cohorts. We leveraged such study design rather than seeking for individual large consortium‐based GWAS for each CVD because of its scalability to conduct a phenome‐wide scan for associations of significant signals with over a hundred of phenotypes. A recent simulation study showed that most of the two‐sample MR methods, including the inverse variance weighted, weighted median and weighted mode MR methods, performed similarly when tested in two independent samples to a single large‐scale biobank, even in the presence of substantial confounding due to sample overlap.^[^ ^79^ ^]^ The bias of MR Egger estimates toward the confounded observational association increases with the magnitude of sample correlation, therefore caution is needed when interpreting results that are only significant in the MR Egger analysis.

## 5 Conclusions

Our study evaluated causal relationships between ≈3000 circulating proteins and 19 CVD using biobank‐scale genetic association summary statistics for proteins and phenotypes in a bi‐directional MR framework. This study provides human genetics‐based evidence of novel candidate genes, a foundational step toward full‐scale causal human biology‐based drug discovery for CVD.

## Conflict of Interest

All authors are employees and/or stockholders of Bristol‐Myers Squibb.

## Author Contributions

C.L., J.C.M., E.M.K., and E.R.H. designed study. C.L. performed the MR and colocalization analyses and N.D.J. performed the single cell integration analyses and visualization. C.L. and N.D.J. wrote the manuscript. All the authors reviewed the manuscript and provided constructive comments.

## Peer Review

The peer review history for this article is available in the Supporting Information for this article.

## Supporting information



Supporting Information

Supplementary Information: Record of Transparent Peer Review

Supporting Information

## Data Availability

The GWAS summary statistics data used in the MR analyses are available from previous publications. Specifically, plasma proteomics data can be accessed via registry of open data on Amazon web services at https://registry.opendata.aws/ukbppp/,^[^ ^20^ ^]^ and CVD data from GWAS Catalog at https://www.ebi.ac.uk/gwas/ with study accession IDs from GCST90018563 to GCST90019002 from Sakaue S. et al.^[^ ^31^ ^]^ All data that support the plots within this paper and other findings of this study are available in the supplementary tables.
