# Supplementary material for: Proteome‐Wide Mendelian Randomization Identifies Candidate Causal Proteins for Cardiovascular Diseases
Source: Adv Genet (Hoboken). 2025 Mar 10;6(2):2500003. doi: 10.1002/ggn2.202500003 (PMC12245532; doi:10.1002/ggn2.202500003)
Supplement: Supplementary file 1 — Supporting Information [file GGN2-6-2500003-s003.docx]

**Extended data figures for: Proteome-wide Mendelian randomization identifies candidate causal proteins for cardiovascular diseases**

Chen Li^1^, Nicolas De Jay^1^, Shan-Shan Zhang^2^, Xin Fang^1^, Supriya Sharma^1^, Katrina A. Catalano^1^, Venkatesh Sridharan^2^, Zhaoqing Wang^3^, Lei Zhao^2^, Joseph D. Szustakowski^1^, Ching-Pin Chang^2^, Joseph C. Maranville^1^, Emily R. Holzinger^1^, Erika M. Kvikstad^1^

1. Informatics and Predictive Sciences, Bristol-Myers Squibb, USA
2. Immunology and Cardiovascular Thematic Research Center, Bristol-Myers Squibb, USA
3. Translational Medicine, Bristol-Myers Squibb, USA

February 2025

This file includes extended data figures.


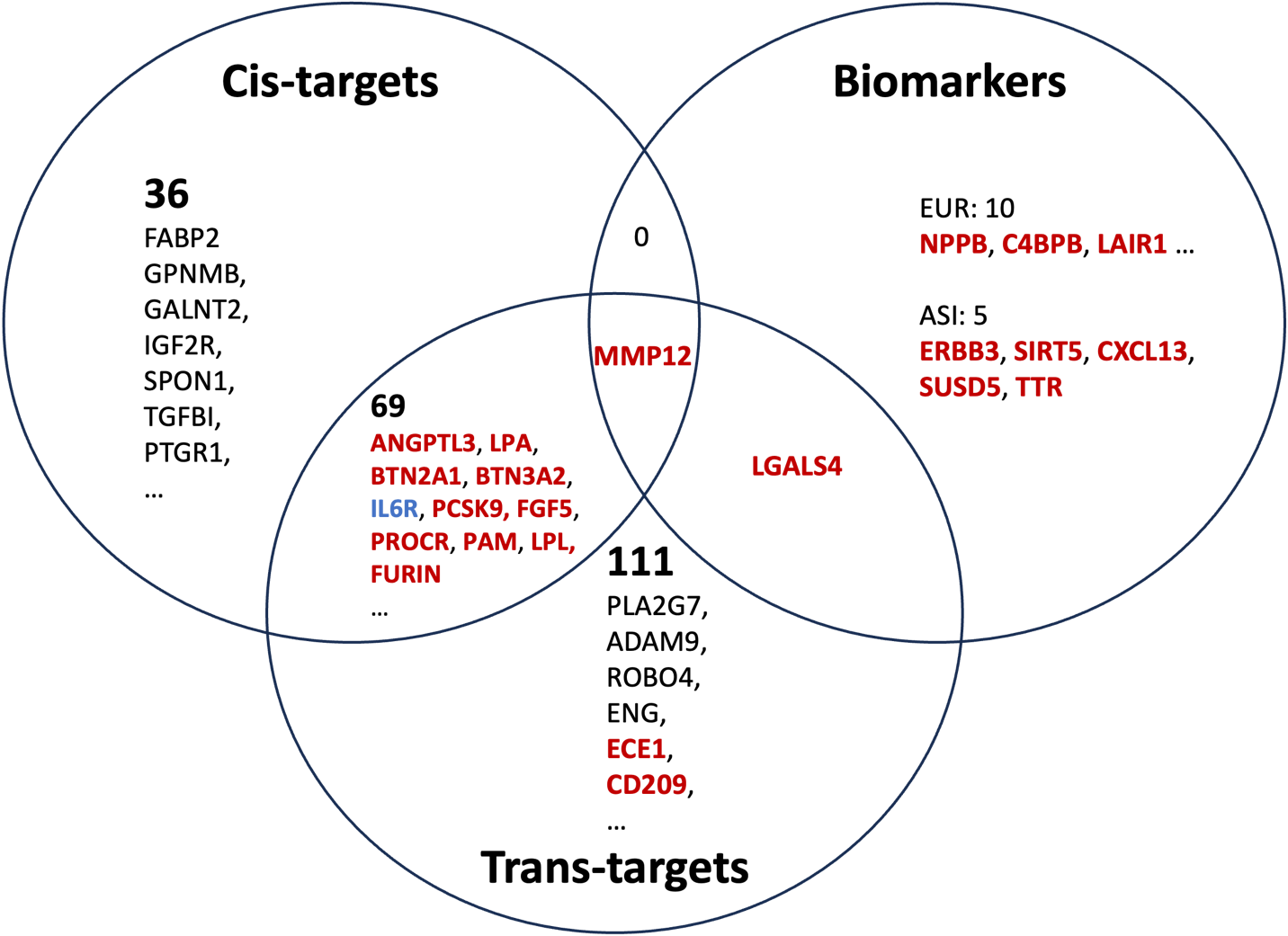


**Figure S1. Venn diagram of shared and distinct proteins identified in forward-MR (cis-pQTLs only for cis-targets and cis- + trans-pQTLs for trans-targets) and reverse-MR (biomarkers) in European (EUR) and Asian (ASI) ancestries.** Cis-targets indicate proteins that are associated with any cardiovascular diseases only through cis-pQTL forward MR analysis, and trans-targets only through trans-pQTL forward MR analysis. Blue color indicates IL6R was identified in both EUR and ASI ancestries, which is the only protein that was identified in both ancestries. There was also no overlap for biomarker findings between EUR and ASI. Red color indicates proteins that were mentioned in the paper.


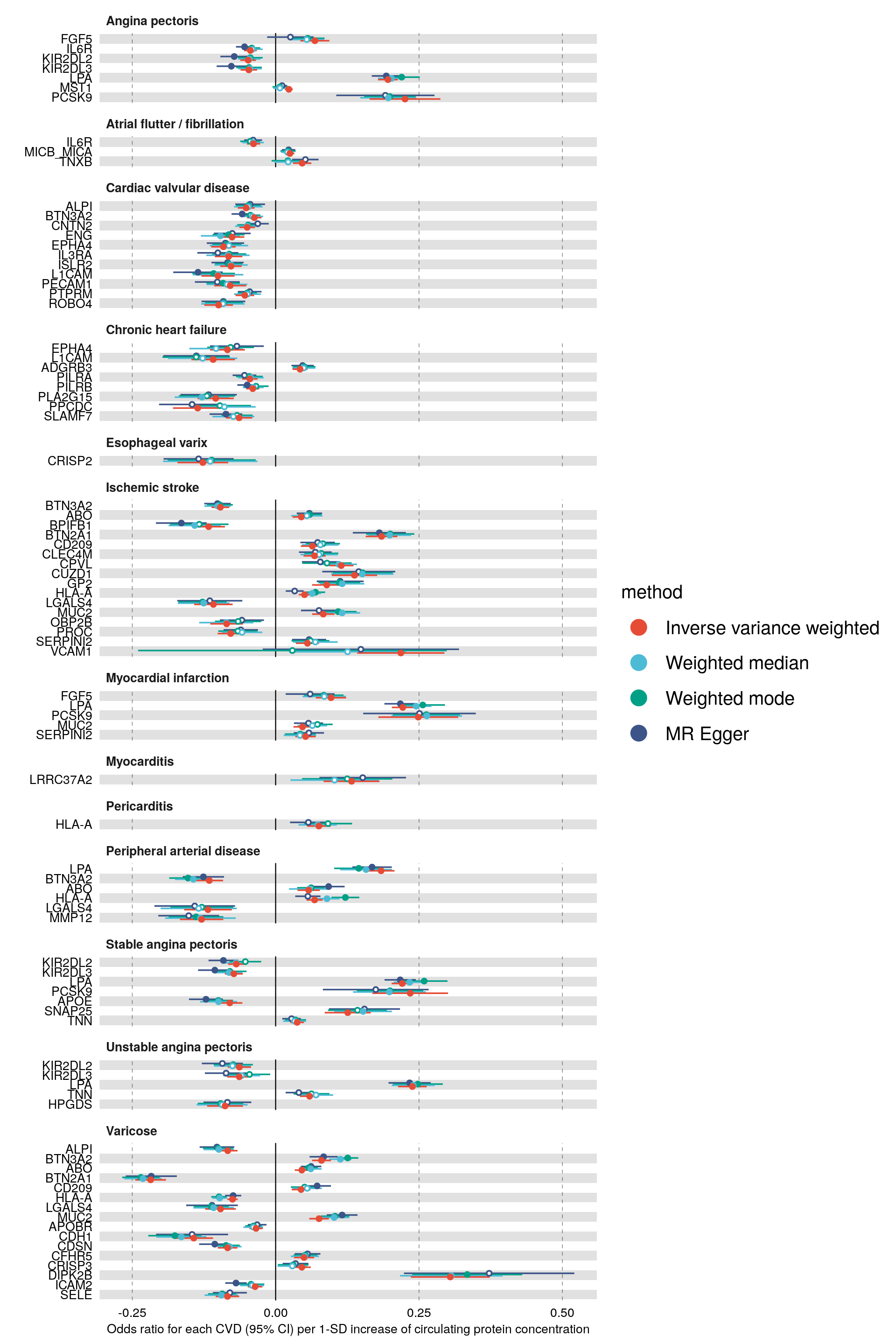

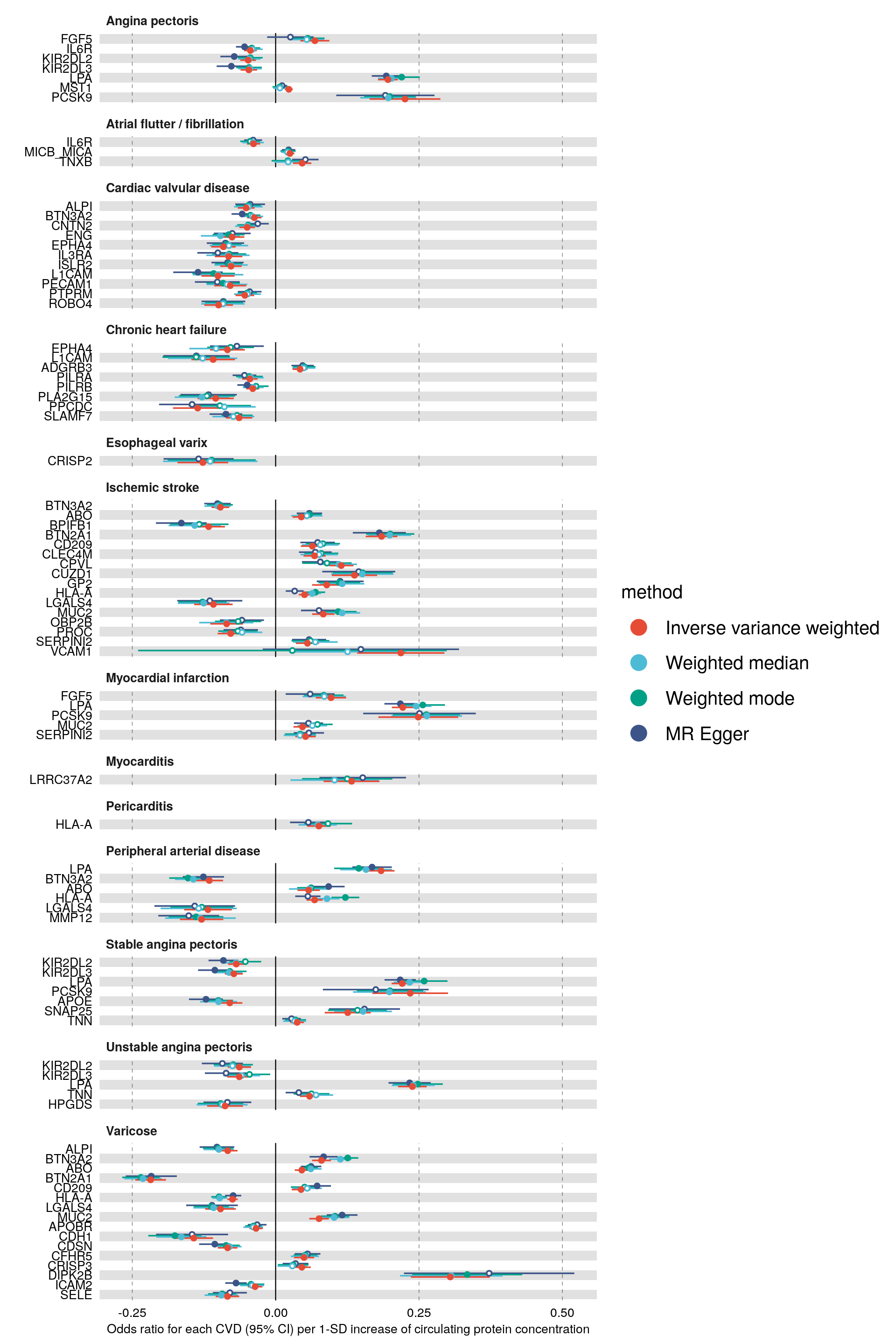


**Figure S2. The full significant results with FinnGen validation across CVD.** Protein effects on CVD were presented as odds ratios (95% CI) per 1-SD increment of circulating protein concentration. Colors indicate 4 different MR methods. Significant results after Bonferroni correction are displayed as filled circles, while non-significant results are shown as hollow circles.

**A.**


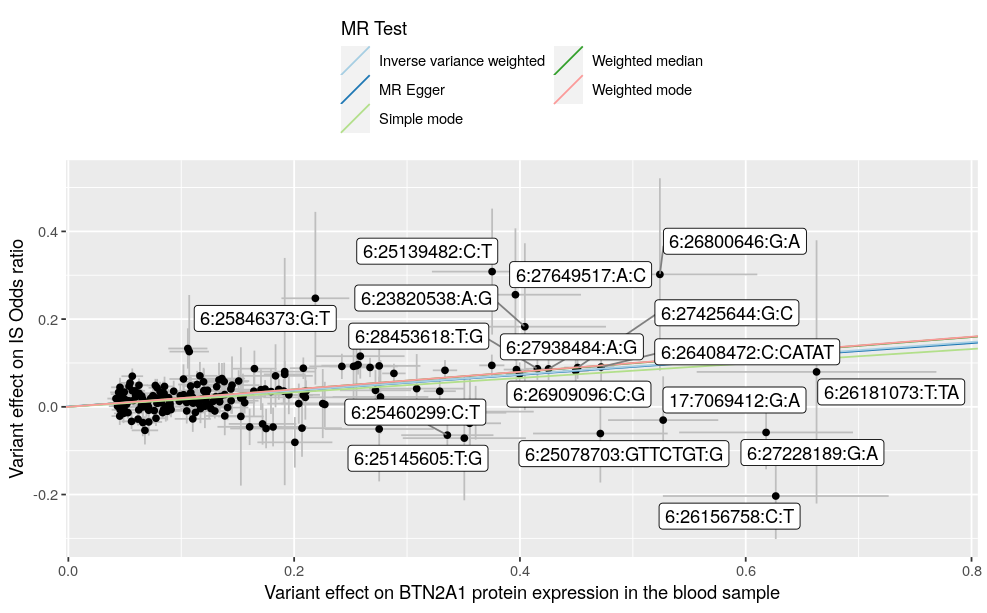


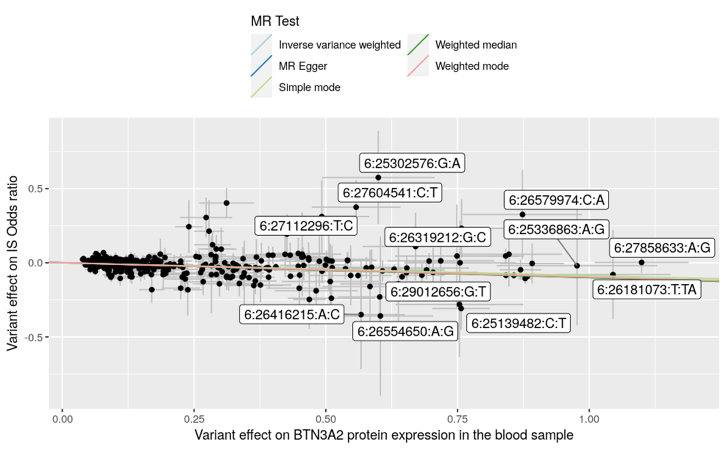
**B.**

**Figure S3. Single variant effects of novel targets on ischemic stroke.** The scatter plot shows single variant effects of **A.** BTN2A1 and **B.** BTN3A2 on their corresponding protein levels and on ischemic stroke. Higher BTN2A1 protein concentration leads to a higher risk of ischemic stroke, while BTN3A2 exerts a protective effect. Variants with the largest effects on protein concentrations are labeled with their chromosome positions and alleles.

A.

**
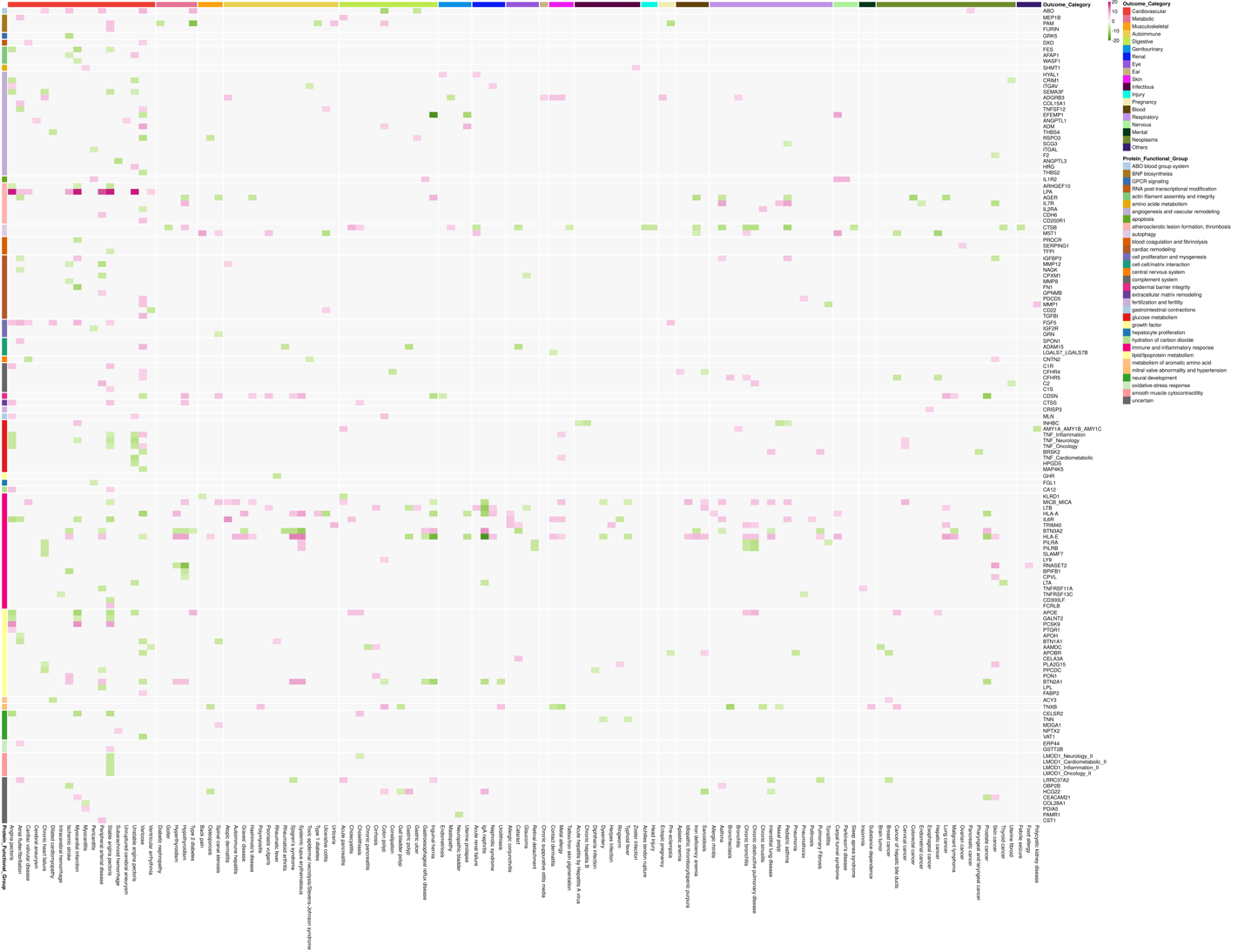
**

**B.**

**
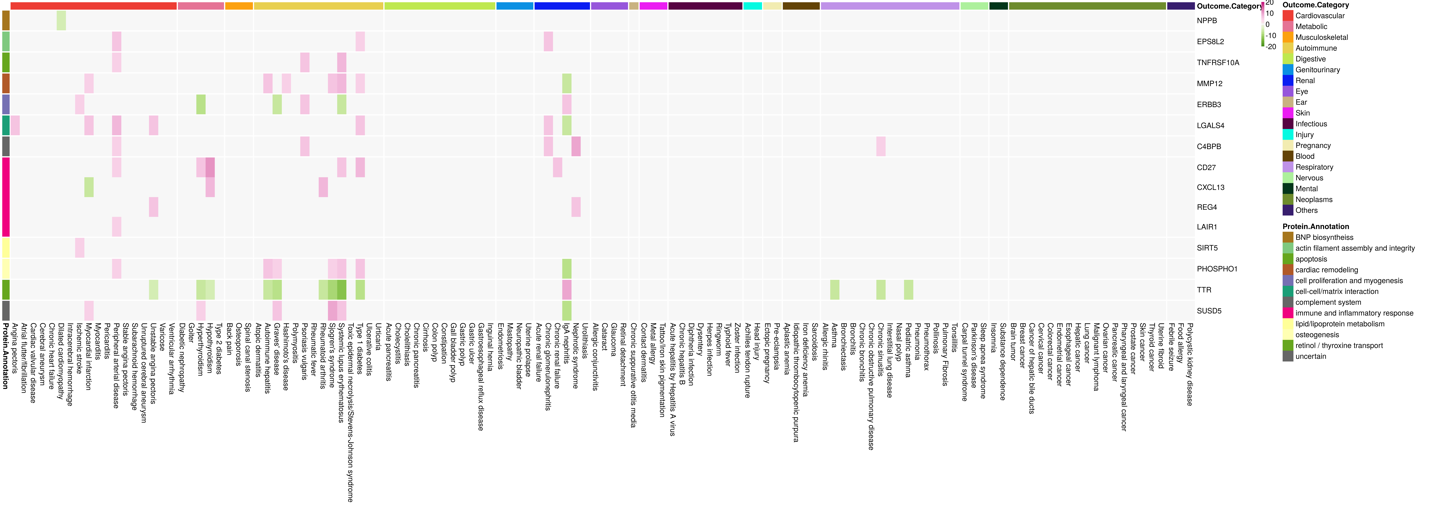
**

**Figure S4. Phenome-wide MR scan to evaluate target and biomarker specificity and pleiotropy.** Heatmap representation of Z-scores from A. forward-MR results using cis-pQTLs as instrumental variables and B. reverse-MR results, with inverse variance weighted method in the European ancestry. For Z-scores of absolute values > 20, they are truncated to a maximum absolute value of 20. The color gradient, from green (negative) to red (positive), illustrates the direction and magnitude of causal associations between proteins (Y-axis) and diseases (X-axis). Diseases and proteins are grouped by their different categories and labeled with different colors on the top and the left-side of the heatmap, respectively.

**A.**


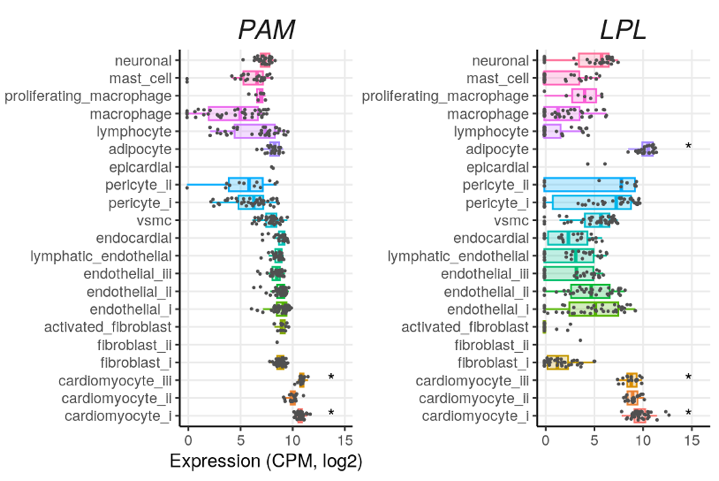


**B.**


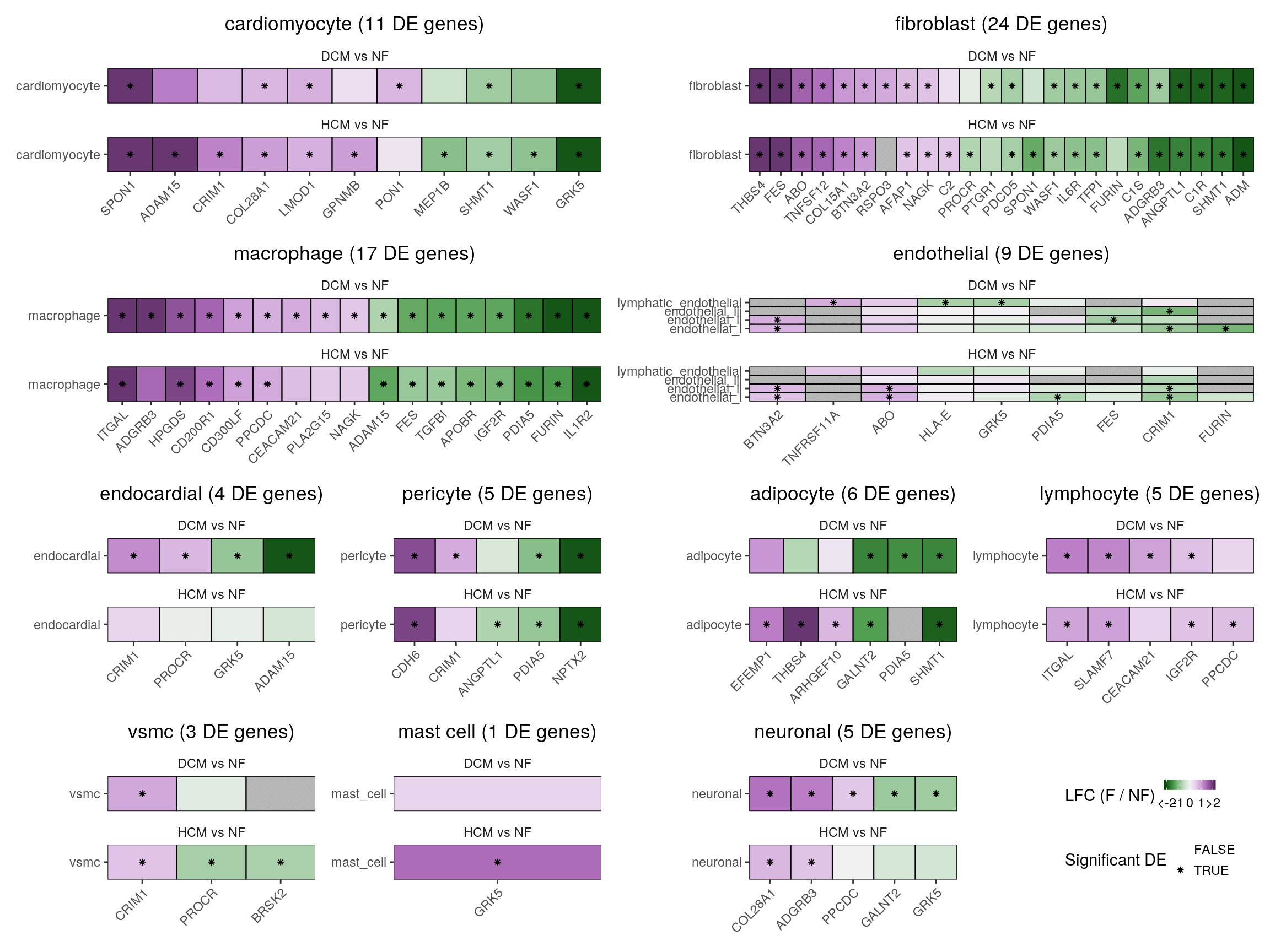


**Figure S5. Cardiac Cell Type-Specific Gene Expression in Cardiomyopathy. A.** Box plots illustrating expression of *LPL* and *PAM* across cardiac cell types. Each point represents one of 42 donors (DCM: n=11, HCM: n=15, NF: n=16). Box plots show the median, IQR (box), and whiskers extending to 1.5x IQR. Asterisks highlight significant enrichment of gene in a cell type relative to the others, using limma-voom. Gene expression is reported in log_2_ counts per million (CPM). **B.** Differential gene expression in DCM or HCM vs non-failing heart samples stratified by cell types. The log_2_ fold change (LFC) of gene expression is scaled to center at 0 and capped to a range of –2 to 2. Asterisks denote significant differential gene expression. Significance is determined using limma-voom.
