# Supplementary material for: Proteome‐Wide Mendelian Randomization Identifies Candidate Causal Proteins for Cardiovascular Diseases
Source: Adv Genet (Hoboken). 2025 Mar 10;6(2):2500003. doi: 10.1002/ggn2.202500003 (PMC12245532; doi:10.1002/ggn2.202500003)
Supplement: Supplementary file 2 — Supplementary Information: Record of Transparent Peer Review [file GGN2-6-2500003-s001.docx]

Record of Transparent Peer Review

Review timeline: Data submitted: 10-Feb-2025

1st Editorial Decision: 12-Feb-2025

Revision Received: 18-Feb-2025

Accepted: 23-Feb-2025

Submission with reports and responses: 10-Feb-2025

**Response to editors’ comments**

We’re grateful for your constructive feedback on the manuscript. Your comments were very helpful and have guided us in improving the manuscript, and we have made the revisions accordingly and listed the specific changes to answer the editors’ comments as follows.

*In general, we agree with the reviewers that the work would be of interest to the broad audience of Nature Cardiovascular Research. At the same time, the review process identified several opportunities to strengthen the work. We would be happy to look at a revised manuscript that has been revised accordingly (unless something similar has been accepted at Nature Cardiovascular Research or appeared elsewhere in the meantime). More specifically, it would be particularly important to us that you address all methodological points raised by Reviewers #1 and 2 and reference the publications they indicate as guidelines. Both referees expressed concerns that your current analysis may not have completely accounted for* ***horizontal pleiotropy****, and we agree with their assessment that this is fundamental. Reviewer #1 further suggests that you place your analysis in the context of previous literature by investigating how many targets colocalise with eQTLs identified in other studies,* ***researching their potential cell of origin*** *based on published datasets, and validating some of the biomarkers identified by* ***reverse-MR*** *in independent plasma protein data. We believe the manuscript would benefit from such improvements and that* ***some of the newly identified genes should be functionally validated****, per Reviewer #1's indications. Although referees #1 and #2 have a different view about the* ***reverse MR*** *data and conclusions, editorially we would prefer that you expand the data along the lines outlined by Referee #1, rather than take them out. We hope that by adding new data and discussion, Reviewer #1 might see the benefits of this approach in the revised study. Although the above points are the most important to us, please do also respond to the other points raised by our referees, ideally with new experiments. If you have any questions or would like to discuss the scope of the revision please do get in touch.*

1. To better account for **horizontal pleiotropy**, we added the sensitivity analyses using different MR approaches, including MR-PRESSO, GSMR and a multi-variable MR method – MRBEE. Most of the significant associations remained robust in sensitivity analyses correcting for horizontal pleiotropy (86% and 92% reached Bonferroni-adjusted P-value threshold after outlier filtering using MR-PRESSO and GSMR methods), with full results shown in the supplementary tables 11-12. We also described the methods and results separately for findings using cis-pQTLs only and cis- and trans-pQTLs as instrumental variables, and replication analyses were performed for cis-pQTLs only and cis- and trans-pQTLs separately as well.
2. To research potential **tissue and cell origin** of newly identified targets, we firstly cited the work and incorporated their results in our manuscript for some of the targets we highlighted, e.g. cis-pQTLs for the BTN3A1 gene were colocalized with eQTLs in the heart left ventricles and various anatomical regions of the brain, and cis-pQTLs for the BTN2A1 gene were colocalized exclusively with eQTLs in the heart left ventricles, which were in line with our findings for their roles in ischemic stroke. Secondly, we further analysed potential colocalizations between 1) a pair of CVD and gene expression at transcriptional level in each tissue from GTEx (v8) 2) a pair of CVD and plasma protein level, as well as 3) a triad of CVD, plasma protein and tissue-specific gene expression levels using HyPrColoc methodology.^1^ HyPrColoc can test statistical colocalization of multiple traits, when testing for two traits, it’s equivalent to coloc.^2^ The full results are presented in the supplementary table 15 and summarized into a paragraph in the Results section of integration of bulk and single cell RNASeq.
3. We validated the biomarkers identified by the **reverse-MR** using FinnGen Olink proteomics data, and in which proteins measured in the FinnGen cohort were used as outcomes. The full results are shown in the supplementary table 10. In brief, four of the 11 biomarkers identified in the European population (MMP12, LGALS4, C4BPB and LAIR1) were replicated using FinnGen Olink proteomics data (P-value < 0.05), and C4BPB reached FDRBH-adjusted significance threshold.” We expanded the discussion part for “the values and limitations of bidirectionality in MR analyses”. The additional analysis and discussion would hopefully help to convince the Reviewer #2 the values in conducting the reverse-MR.
4. We discussed with the editors and agreed that **experimental validation** may be out of scope for this manuscript. To strengthen our evidence for biological function of novel targets, we performed 1) in-depth literature review for functional experiments already undertaken, and 2) an *in-silico* validation experiment to knock in (overexpression) or knock out (downregulation) genes in human cardiomyocytes using a deep learning model called Geneformer.^3^ Details are specified in the response to Reviewer #1’s fourth comment.

**Response to reviewers’ comments:

Reviewer #1**

*1.* ***eQTLs vs pQTLs colocalization****. The idea of plasma protein quantitative traits (ppQTLs) is somewhat troublesome to me. As opposed to the inference of tissue eQTLs (or pQTLs for that matter) where the genetic regulation of genes in cells of a given tissues is sought, plasma does not contain cells. Hence, ppQTLs should by nature be indirect reflections of tissue eQTLs (or cell-specific eQTLs). It is therefore appreciated the authors to some extent try to find the source tissue/cells for the ppQTLs judged to be causal for a range of CVDs in their fwd MR analysis. However, colocalization analysis using extensive eQTLs databases like GTEx or the more CVD-relevant, STARNET databases is an intermediate step completely lacking in the current study.* ***I suggest after the initial MR exploration using UK biobank, the authors should, before the validation in FInnGen; investigate how many of the “targets” colocalize with eQTLs identified for the corresponding genes in these studies.*** *In large, these analyses have already been performed in the original UK biobank Nature paper 2024. Thus, should be a relatively easy thing to explore.*

We appreciate the Reviewer’s comment. The pairwise colocalization analyses have been performed to identify shared genetic associations between variants affecting each circulating protein and its corresponding tissue-level gene expression in the UKB-PPP flagship paper^4^. The analyses have assessed all genes measured in the Olink proteomics panel that have at least one significant pQTL and eQTL, and our identified targets are among these assessed genes. In a total of 113 targets identified using cis-pQTLs, 73 genes had shared causal variants for their associations with plasma protein levels and transcriptional gene expression levels in at least one tissue. **We cited the work and incorporated their results in our manuscript for some of the targets we highlighted,** e.g. cis-pQTLs for the BTN3A2 gene were colocalized with eQTLs in the heart left ventricle and various anatomical regions of the brain, and cis-pQTLs for the BTN2A1 gene were colocalized exclusively with eQTLs in the heart left ventricle, which were in line with our findings for their roles in ischemic stroke. **Moreover, we further analysed potential colocalizations between 1) a pair of CVD and gene expression at transcriptional level in each tissue from GTEx (v8) 2) a pair of CVD and plasma protein level, as well as 3) a triad of CVD, plasma protein and tissue-specific gene expression levels using HyPrColoc methodology.^1^** HyPrColoc can test statistical colocalization of multiple traits, when testing for two traits, it’s equivalent to coloc.^2^ The full results are presented in the supplementary table 15 and summarized into a paragraph in the Resultd section of integration of bulk and single cell RNASeq.

*2.* ***Horizontal pleiotropy*** *occurs when the variant influences disease outside of its effect on the exposure in MR. Violation of the ‘no horizontal pleiotropy’ assumption can cause severe bias in MR. I am therefore doubtful to use trans pQTLs, and just stating they need follow-up is simply not good enough. At a minimum, the authors should perform something like what is described in this article:* [*https://www.ncbi.nlm.nih.gov/pmc/articles/PMC6083837/*](https://www.ncbi.nlm.nih.gov/pmc/articles/PMC6083837/)

Thank you for the insightful comments. **To test horizontal pleiotropy, we conducted additional analyses using MR-PRESSO^5^ and GSMR^6^ methods.** The MR-PRESSO detects the horizontal pleiotropy by comparing observed residual sum of squares (the overall differences between observed and predicted effects of variants on the outcome) to expected residual sum of squares simulated under the null hypothesis of no horizontal pleiotropy. It estimates a corrected causal effect by removing horizontal pleiotropic outlier variants. The full results are shown in supplementary table 12. In brief, 108 out of the total 348 significant pairs of associations from the forward MR analysis using cis- and trans-pQTLs as instrumental variables showed at least one significant outlier, among these 93 (86%) pairs reached Bonferroni-corrected P-value threshold (P-value <= 1.27e-7) after outlier correction, and all the associations reached FDR-adjusted significance threshold of 0.05.

The GSMR method can correct horizontal pleiotropy by removing HEIDI-outliers (variants that show significant differences in causal estimates from the top exposure-associated SNP). The full results are shown in supplementary table 13. In brief, there were 345 pairs of significant protein-to-disease associations analysed (proteins with at least 5 genetic instrumental variables, because the GSMR method requires quintiles of causal estimate distribution to define pleiotropic outliers), among which 238 pairs showed at least one pleiotropic outlier (HEIDI < 0.01) and 219 (92.0%) pairs reached Bonferroni-corrected P-value threshold after filtering the outliers, and all pairs reached FDR < 0.05.

These two additional sensitivity analyses demonstrated that most of IVW-based significant results remain robust in the sensitivity analyses that correct for horizontal pleiotropy, and we mentioned these results in the “Target discovery with forward-MR” result section.

*3. I am also curious how often* ***trans-pQTLs are shared between proteins****, as using the same instruments for different exposures without combining them in* ***a multivariate MR*** *does not, according to me, make sense. Please clarify your approach and be more transparent about these results. Also perform multivariate MR when necessary.*

Thank you for raising this question and the invaluable comments. We **assessed the probabilities when genetic instruments, i.e. pQTLs, were shared between proteins**. The majority (88.2%) of genetic instruments were associated with only one protein for the 188 targets identified using cis- and trans-pQTLs combined, 345 out of 28,688 (1.2%) pQTLs were associated with at least 5 proteins, and 11 pQTLs – highly pleiotropic were associated with at least 30 proteins. Multivariable MR can estimate the direct causal effect of each protein independent of other proteins associated with horizontally pleiotropic variants. **We applied MRBEE^7^, a cutting-edge multivariable MR method that eliminates weak instrument bias while accounting for horizontal pleiotropy, for all significant proteins using cis- and trans-pQTLs combined for each cardiovascular disease outcome.** The full results are illustrated in the supplementary table 14. In brief, 20 CVDs associated with more than one significant protein were analyzed (347 out of 348 significant pairs). In the 347 significant pairs, 66 remained nominally significant (32 pairs with FDR < 0.05) in the multivariable MR adjusting for all other significant proteins associated with a disease. It's known that multivariable MR methods designed for high-dimensional settings are often biased towards null in previous simulation studies,^8^ therefore the power of identifying true signals is less than using the IVW method, and there may be mediation and confounding effects among proteins, with multivariable MR independent causal effects can be estimated. Some signals with strong biological evidence remained highly significant in the multivariable MR, including some we highlighted in vignettes, e.g. LPA for angina, cardiac valvular disease and cardiomegaly, ADAM23 for cardiomegaly, BTN1A1 for atrial fibrillation and PAM for ventricular arrhythmia.

*4. The authors propose several novel targets for CVDs - some experimental validation would be becoming.*

We appreciate the reviewer’s comments. While we think having such wet lab based experimental validation would help strengthen the work, it may be slightly out of scope for the current manuscript. We also noticed that there were recent statistical genetics / Mendelian randomization work published in Nature family journals without wet lab experiment validation, for example, Lingyan Chen et al. Nat Commun. 2022 (PMID: 36253349)^9^, Sonia Shah et al. Nat Commun. 2020 (PMID: 31919418)^10^ and Jie Zheng et al. Nat Genet. 2020 (PMID: 32895551)^11^. **We strengthened our evidence for biological function of novel targets by 1) literature review for functional experiments already undertaken.** For example, in vitro and in vivo studies showed ADAM23 protects against cardiac hypertrophy possibly through focal adhesion kinase-protein kinase B signaling pathway, which has been established as a key component activated by integrins. Integrins are located in Z-discs and intercalated discs in cardiac myocytes, providing a mechanical linkage that enables coordinated contraction of cardiomyocytes (line 367-280). **2) we performed an *in-silico* validation experiment to knock in (overexpression) or knock out (downregulation) genes in human cardiomyocytes using a deep learning model called Geneformer.^3^** We finetuned the pretrained model from the Geneformer paper on 3 datasets that contain data for HCM and DCM human patients^12,13^ and a DCM pre-clinical model.^14^ Simulation of gene knockout or overexpression was performed for one gene at a time following the direction predicted by MR, and we tested if the perturbation significantly moved the cell from disease to healthy states. Out of 37 genes tested that are associated with DCM, HCM, Cardiomegaly and Chronic heart failure in the MR analysis, 14 genes were predicted to significantly move cells from disease to healthy states shown in the table below. There are some limitations for this method of validation, e.g. genes are expressed in the cardiomyocytes, and the performance of this model is limited by the single cell data sample sizes and the number of cardiomyocytes used for finetuning.

| **Gene**  **Name** | **Shift from disease to healthy** | **p-value FDR** | **Dataset** | **Disease** | **Perturbation** |
| --- | --- | --- | --- | --- | --- |
| **LRPAP1** | 6.20E-04 | 2.92E-02 | Liu | HCM | Knockout |
| **ABHD14** | 3.06E-03 | 1.18E-08 | Huang | DCM_ipsc_cardiomyoctes | Knockout |
| **SGSH** | 2.29E-03 | 5.23E-04 | Huang | DCM_ipsc_cardiomyoctes | Knockout |
| **LRPAP1** | 2.13E-03 | 2.46E-09 | Huang | DCM_ipsc_cardiomyoctes | Knockout |
| **LRP1** | 4.87E-03 | 1.13E-09 | Chaffin | HCM | Knockout |
| **MET** | 9.94E-03 | 2.62E-05 | Liu | HCM | Overexpression |
| **GYS1** | 6.49E-03 | 1.74E-09 | Liu | HCM | Overexpression |
| **CD58** | 4.65E-03 | 4.42E-03 | Huang | DCM_ipsc_cardiomyoctes | Overexpression |
| **CTSB** | 2.94E-03 | 1.73E-02 | Chaffin | DCM | Overexpression |
| **PPCDC** | 1.20E-03 | 2.17E-02 | Chaffin | DCM | Overexpression |
| **PILRB** | 7.77E-04 | 2.57E-03 | Chaffin | DCM | Overexpression |
| **GYS1** | 7.00E-06 | 4.51E-03 | Chaffin | DCM | Overexpression |
| **CD34** | 5.97E-03 | 2.11E-02 | Chaffin | HCM | Overexpression |
| **THBS4** | 2.90E-03 | 1.67E-05 | Chaffin | HCM | Overexpression |
| **PLA2G1** | 2.42E-03 | 1.53E-03 | Chaffin | HCM | Overexpression |
| **CTSB** | 4.53E-03 | 3.83E-08 | Chaffin | HCM | Overexpression |
| **EPHA4** | 3.20E-03 | 9.47E-10 | Chaffin | HCM | Overexpression |

**Minor points**

*1) line 32: ”Genome-wide association studies (GWAS) 30 of circulating proteins identify genetic variants that regulate protein levels in plasma, which can be used as instrumental variables (IVs) to infer causality under the framework of Mendelian randomization (MR).” - as above rephrase to GWAS identifies genetic variants in cells that regulate gene expression which in turn may have effects on the levels of plasma proteins (or similar phrasing).*

Thank you for the reviewer’s suggestion. We agree on the reviewer’s point that the genetic effects on protein plasma levels can be a consequence of genetic effects on cellular protein levels across tissues, and accordingly **changed the sentence to** “Genome-wide association studies (GWAS) of circulating proteins identify genetic variants in cells that regulate protein expression that in turn may have effects on protein levels in plasma, and these genetic variants can be used as instrumental variables (IVs) to infer causality of protein expression on CVD risk under the framework of Mendelian randomization (MR)”.

2*) Methods/Results: The authors should clearly separate their MR analyses using trans vs cis SNPs. Of the target proteins found in UK biobank – how many were a result of trans vs cis pQTLs. As of now this is only shown in part in the validation using FinnGen.*

Thank you for the valuable comment. We **edited the description in the methods and results and clarified that** we assessed cis- and cis- + trans-pQTLs as IVs separately both in the UKBB discovery and FinnGen validation analyses.

*3) There are now several snATAC seq datasets (like PMID: 35590109) which can also be used to examine likely cell type origin of target pQTLs. This would be an important addition to the cell enrichment and DE cell-type specific analyses.*

We appreciate the reviewer's insightful suggestion. We retrieved data from the Kuppe et al. *Nature* 2022 (PMID: 35948637) paper since the authors provided snATAC-seq data from cardiac biopsies that include cardiomyocytes, a highly relevant cell type for heart failure, and that aligns with our choice of transcriptomic snRNA-seq reference.

To examine the likely cardiac cell-type origin of target pQTLs, we associated each lead pQTL to the closest chromatin peak within a 1 kb window and calculated whether chromatin accessibility was significantly enriched in one cell type versus all others.

Methods are specified in detail below. We retrieved processed snATAC-seq data from Kuppe et al. Nature 2022 (PMID: 35948637) using the ArchR (v1.0.2; PMID: 33633365) R package. We ensured adequate representation of each cardiac cell type by selecting high-quality cells from both healthy hearts and non-infarcted regions ("remote zone") of myocardial infacrtion patients. To define chromatin accessibility peaks, we first aggregated insertions from each cell type into pseudo-bulk profiles. Next, we performed peak calling in each pseudo-bulk profile using MACS2 (q-value < 0.05) (v2.2.9.1; PMID: 18798982). To derive a consensus peak set across the pseudo-bulk profiles, we used the ArchR’s iterature overlap peak merging procedure. To identify cell-type-specific peaks, we conducted differential accessibility analysis between cells in each cell type in a one-versus-rest approach using a Wilcoxon test. Peaks demonstrating large (log2 fold-change>1) and statistically significant (FDR<0.01) enrichment were considered to be cell-type-specific.

*4) Line 215: Please re-write this section specifying cis and trans pQTLs clearly “Among
the 221 candidate causal proteins (cis n= ???, trans n=??) , 139 (62.9% (cis n= ???, trans n=++)) were replicated (FDRBH-adjusted P-value < 0.05) using pQTLs from FinnGen Olink analyses, and 76 (34.4%) reached Bonferroni corrected significance threshold (Figures 2-3 and Supplementary Figures 3-4).*

Thanks for the comment. We **rewrote the sentence to explicitly show distinct proteins replicated with cis- + trans-pQTLs and cis-pQTLs only separately**. “There was a total of 107 proteins replicated (FDRBH-adjusted P-value < 0.05, 92 with cis- + trans-pQTLs and 47 with cis-pQTLs only) from the FinnGen Olink analyses, and 58 proteins (56 with cis- + trans-pQTLs and 17 with cis-pQTLs only) reached Bonferroni corrected significance threshold”.

*5) I highly appreciate the marker discovery using reverse-MR. To me it makes more sense that most plasma proteins are a consequence of physiology and disease rather than causes - proteins that are secreted or leaking from disease (physiological) processes into the circulation. The ms would benefit greatly if some or at least one of the identified biomarkers could be validated in independent plasma protein data? Perhaps one of the 5 Asian-specific biomarkers?*

We appreciate the reviewer’s comment. **We conducted additional validation analyses for the biomarkers identified using FinnGen Olink data**, in which proteins measured in the FinnGen cohort were used as outcomes. The full results are shown in the supplementary table 10. We also added “Four of the 11 biomarkers identified in the European population (MMP12, LGALS4, C4BPB and LAIR1) were replicated using FinnGen Olink proteomics data (P-value < 0.05), and C4BPB reached FDRBH-adjusted significance threshold.” in the Results section of biomarker discovery with reverse-MR. For the biomarkers identified in the Asian cohort, we’re restricted by publicly accessible data sources for an independent test in this ancestry.

*6) In the section “Advantage and limitation in integrating single-cell transcriptome with MR study”, the authors state “dynamics of plasma proteome alone cannot fully
543 represent tissue-specific changes of protein profiles(61)” . I have not read the 2007 ref in full but where else would plasma proteome originate from (at least not from plasma itself)? Obviously, it is unlikely that the “seed” gene (i.e., transcription levels of corresponding gene in a single tissue) alone is responsible for a plasma protein but likely post-translation regulation involving many genes across several tissues are likely key for the levels of plasma proteins. Please expand on these thoughts in this section.*

We thank the reviewer for the insightful comment. Plasma protein levels are not solely driven by transcriptional changes in a single tissue. Instead, they result from complex, multi-tissue interactions and regulatory mechanisms, including post-translational modifications, protein transport, and systemic regulation. The tissue-specific transcriptomic profiles we integrate with MR serve as proxies for identifying where these proteins may be primarily regulated, but they are not the sole contributors to the dynamic plasma proteome. **We expanded the discussion further on this idea at line 622-626.**

Reviewer #2 (Remarks to the Author):

*There are substantial issues with these analyses that need addressing:
1. Insufficient detail on the study outcomes.
The authors report MR analyses of 21 ‘cardiovascular disease’ outcomes but I could not find any detail in the manuscript as to how these diseases were defined and how many cases of each disease were analysed. It seems that the cases came from UK Biobank, FinnGen or Biobank Japan. In UK Biobank, disease outcomes may be self-reported or come from linked to National Health Service primary care data or hospital episodes coded using Read, ICD-10 or SNOMED. Were the conditions self-reported or from NHS data and, if so, which codes were used for each disease? Failure to properly report these details makes it impossible for other researchers to verify or reproduce the findings. How were the disease definitions aligned across UK Biobank, FinnGen and Biobank Japan? The authors refer to a previous study (reference 22) in relation to study outcomes, but this is insufficient in my view.*

We thank the review’s thoughtful comments. **We listed the disease definitions and sample sizes in the supplementary table 1 and added more description of the disease definitions in the methods (reverse MR).** For GWAS full summary statistics, we used publicly available data from the paper Sakaue, S. et al.^15^, which can be downloaded here: <https://humandbs.dbcls.jp/en/hum0197-v3-220>. Phenotypes were curated from past medical history and text-mining of electronic medical records data in the Biobank Japan and mapped to phecode and ICD10. UK Biobank disease definition was based on diagnoses from hospital inpatient records (field ID 41270) and the disease endpoints of FinnGen were mapped to those in the Biobank Japan using ICD10 and phecode. IV definition for each CVD was developed based on this GWAS meta-analysis for Europeans and Asians separately. For hypertrophic and dilated cardiomyopathy, IVs were built from a more cardiomyopathy-focused GWAS with greater statistical power for Europeans.^16^ More details of the disease definition were described in these two GWAS papers.

*Other issues:
•One outcome listed is ‘cardiomegaly’ but this is a morphological description not a disease.
• One outcome listed is EV - esophageal varix. Oesophageal varices arise due to portal hypertension as a consequence of chronic liver disease and would not conventionally be considered a cardiovascular end point.*

We thank the reviewer’s comments. The 21 CVD were selected **based on the ICD10 codes for the category of “diseases of the circulatory system”,** starting with the letter “I”. Cardiomegaly is coded as I51.7 under the ICD10 code classification of “complications and ill-defined descriptions of heart disease”. Esophageal varix is coded as I85, although cirrhosis and other liver diseases are common causes of the disease, it is manifested as enlarged veins at esophagus, and often treated with beta-blocker. We admit that there may be some diseases on the borderline of being classified as CVD or can be categorized into other disease categories if using different classification systems, ICD10 code-based disease classification system is one of the widely used system to classify medical diagnoses worldwide, which is organized based on human body anatomical structure and clinical manifestations.

*2. Problems with the design of the Mendelian randomisation analysis
There are three main issues with the MR analyses: (a) the overall design of the forward-MR studies; (b) the selection of genetic instruments; and (c) the ‘reverse MR’ analyses.
(a) Overall design
UK biobank was the source of the genotype-protein effect estimates as well, it seems, as a source of the genotype-disease outcome estimates making this a one-sample MR design, which is suboptimal. Many of the disease outcomes evaluated (e.g. coronary heart disease, heart failure and atrial fibrillation) have been the subject of very large-scale metaGWAS, which means that the summary outcome effect estimates from these GWAS could have been used in an optimal two-sample design, with much greater power. The authors need to provide much greater clarification of the MR analysis design, conduct a two-sample analysis where the data are available or provide an explanation as to why they do not take a two-sample approach.*

We thank the reviewer’s insightful comments. The CVD GWAS summary statistics were retrieved from one large-scale meta-analysis of UKBB + FinnGen + BBJ, and we analysed separately for Europeans (UKBB + FinnGen) and Asians (BBJ). GWAS summary statistics for plasma proteins were from a subset of UKBB, therefore, the reviewer is correct that there are sample overlaps in the genetic associations with the exposures and the outcomes. We used this study design mainly because of 3 reasons: 1) Many large consortia-based (e.g. CARDIoGRAMplusC4D) GWASes include UKBB, and not necessarily provide larger sample sizes than UKBB + FinnGen, and some don’t make full summary statistics publicly available without restrictions. 2) It’s more scalable, and in this study, we included a phenome-wide scan for significant signals, which was enabled with such pan-GWAS datasets that systematically analysed hundreds of phenotypes. 3) a recent simulation study shows that even in the presence of substantial correlations due to sample overlaps, most of the two-sample MR methods we used in one sample – a large-scale biobank performed similarly to when used in two samples. Therefore, the study concluded that most of the two-sample MR methods can be safely used for one-sample MR performed within large biobanks.^17^ **We summarized the above into limitations in the discussion part “other limitations”.**

**Moreover, we added a multivariable MR analysis using a very recent method -- MRBEE that can solve sample overlapping bias.**^7^ The results were added in the target discovery with forward MR section and full results were shown in the supplementary table 14.

*(b) Selection of genetic instruments
The authors utilise trans- as well as cis instruments for the MR analysis, despite the fact that it has been argued and shown that trans-instruments for protein exposures in MR analysis are prone to horizontal pleiotropy, leading to misleading causal inference. See the following references and the figure taken from the Cold Spring Harbor perspective):*

*Swerdlow DI, Kuchenbaecker KB, Shah S, Sofat R, Holmes MV, White J, Mindell JS, Kivimaki M, Brunner EJ, Whittaker JC, Casas JP, Hingorani AD. Selecting instruments for Mendelian randomization in the wake of genome-wide association studies. Int J Epidemiol. 2016 Oct;45(5):1600-1616.
Schmidt AF, Finan C, Gordillo-Marañón M, Asselbergs FW, Freitag DF, Patel RS, Tyl B, Chopade S, Faraway R, Zwierzyna M, Hingorani AD. Genetic drug target validation using Mendelian randomisation. Nat Commun. 2020 Jun 26;11(1):3255. 
Henry A, Gordillo-Marañón M, Finan C, Schmidt AF, Ferreira JP, Karra R, Sundström J, Lind L, Ärnlöv J, Zannad F, Mälarstig A, Hingorani AD, Lumbers RT; HERMES and SCALLOP Consortia. Therapeutic Targets for Heart Failure Identified Using Proteomics and Mendelian Randomization. Circulation. 2022 Apr 19;145(16):1205-1217. 
Schmidt AF, Hingorani AD, Finan C. Human Genomics and Drug Development. Cold Spring Harb Perspect Med. 2022 Feb 1;12(2):a039230.*

*The authors compare and contrast cis and trans instruments in MR analysis (lines 479-484) but appear to attach equal emphasis to both which is, in my view, incorrect.*

This comment was also raised by another reviewer. We thank the reviewer’s thoughtful comments, and here is my response to the horizontal pleiotropy question. **To test horizontal pleiotropy, we conducted additional analyses using MR-PRESSO^5^ and GSMR^6^ methods.** The MR-PRESSO detects the horizontal pleiotropy by comparing observed residual sum of squares (the overall differences between observed and predicted effects of variants on the outcome) to expected residual sum of squares simulated under the null hypothesis of no horizontal pleiotropy. It estimates a corrected causal effect by removing horizontal pleiotropic outlier variants. The full results are shown in supplementary table 12. In brief, 108 out of the total 348 significant pairs of associations from the forward MR analysis using cis- and trans-pQTLs as instrumental variables showed at least one significant outlier, among these 93 (86%) pairs reached Bonferroni-corrected P-value threshold (P-value <= 1.27e-7) after outlier correction, and all the associations reached FDR-adjusted significance threshold of 0.05.

The GSMR method can correct horizontal pleiotropy by removing HEIDI-outliers (variants that show significant differences in causal estimates from the top exposure-associated SNP). The full results are shown in supplementary table 13. In brief, there were 345 pairs of significant protein-to-disease associations analysed (proteins with at least 5 genetic instrumental variables, because the GSMR method requires quintiles of causal estimate distribution to define pleiotropic outliers), among which 238 pairs showed at least one pleiotropic outlier (HEIDI < 0.01) and 219 (92.0%) pairs reached Bonferroni-corrected P-value threshold after filtering the outliers, and all pairs reached FDR < 0.05.

These two additional sensitivity analyses demonstrated that most of IVW-based significant results remain robust in the sensitivity analyses that correct for horizontal pleiotropy, and we mentioned these results in the “Target discovery with forward-MR” result section.

In addition, **we rewrote relevant results for cis-pQTLs only and cis- + trans-pQTLs as IVs separately both in the UKBB discovery and FinnGen validation analyses**. We edited and highlighted pros and cons for each.

*(c) ‘reverse-MR’ analysis
The authors place emphasis on reverse MR analysis in which the disease is the exposure and the protein of interest the outcome. Where evidence is obtained that the disease alters the protein rather than vice versa they term such proteins ‘biomarkers’ and suggest their approach is useful for biomarker discovery. However, the conventional definition of a biomarker is a biological variable associated with a disease. More biomarkers could be discovered simply by assessing the observational associations of the 3000 or so proteins with the 21 diseases, regardless of any cause effect relationship. Biomarkers that are not causal may still be useful in disease prediction. In my view the ‘reverse-MR’ analyses are not useful and could be dropped.*

We appreciate the reviewer’s comment. We agree that biomarkers can be defined in different ways depending on different context, and our biomarker definition here is to distinguish it from target. The proteins found in an observational association may be called biomarkers in a broader sense (not necessarily causal and with uncertain directionality), and the biomarkers in this study refer to a narrower sense of definition – proteins whose plasma levels are causally influenced by CVD. **We clarified our biomarker definition in the manuscript.** **Moreover, we conducted additional validation analyses for the biomarkers identified using FinnGen Olink data**, in which proteins measured in the FinnGen cohort were used as outcomes. The full results are shown in the supplementary table 10. We also added “Four of the 11 biomarkers identified in the European population (MMP12, LGALS4, C4BPB and LAIR1) were replicated using FinnGen Olink proteomics data (P-value < 0.05), and C4BPB reached FDRBH-adjusted significance threshold.” in the result section of biomarker discovery with reverse-MR. For the biomarkers identified in the Asian cohort, we’re restricted by publicly accessible data sources for an independent test in this ancestry. Furthermore, we expanded the discussion part for “the values and limitations of bidirectionality in MR analyses” from line 505-515, e.g. previous MR studies also included the reverse-MR to assess bi-directional causalities.^11^ Another reviewer raised an opposite opinion on the reverse-MR and suggested it is important and valuable to have. We hope the additional analysis and discussion would help support the value of conducting the reverse-MR.

*3. Permissions, disclosures and funding source
Research in UK Biobank is subject to project approval. I could see no reference to a project approval. It is possible that this work was done as part of the UK Biobank Pharma Proteomics project in which case that should be stated along with details of any preferential or early access to data that made this work possible. I could also see no acknowledgement of funding. The UK Biobank has a funding page (*[*https://www.ukbiobank.ac.uk/learn-more-about-uk-biobank/about-us/our-funding*](https://nam02.safelinks.protection.outlook.com/?url=https%3A%2F%2Furldefense.com%2Fv3%2F__https%3A%2Fwww.ukbiobank.ac.uk%2Flearn-more-about-uk-biobank%2Fabout-us%2Four-funding__%3B!!NLFGqXoFfo8MMQ!raG-g30ZJ01Sws2eCMnHc5nJdmwaaTDFbVDKCiCX7PB3NcisdKzGDd_TQ0LYbWMqNF7WVisk96w60FIWh8uhryD8MALag0h4G2I%24&data=05%7C02%7Cchen.li%40bms.com%7Ca224873368b545b6c27708dcafd3c59b%7C71e34cb83a564fd5a2594acadab6e4ac%7C0%7C0%7C638578571305563540%7CUnknown%7CTWFpbGZsb3d8eyJWIjoiMC4wLjAwMDAiLCJQIjoiV2luMzIiLCJBTiI6Ik1haWwiLCJXVCI6Mn0%3D%7C0%7C%7C%7C&sdata=iIhQ3sDlHK2rQ%2FljUIo%2BqumdI5gtneFw%2FvcR89Jg9f0%3D&reserved=0)*) which multiple sources of UK taxpayer and charitable funding which should be properly acknowledged. Similar statements might also be available for FinnGen and UK Biobank. If, linked electronic health record data was used acknowledgement should be given to the UK National Health Service which provides health care free at the point of delivery funded through general taxation. 
Given the authors are all employees and stockholders of BMS, can they provide reassurance that all findings made as part of this analysis are disclosed in this publication and that none have been held back for commercial reasons.*

Thank you for the review’s reminder. We added the acknowledgements section according to the review’s comments.

*Reviewer #3 (Remarks to the Author):

This is generally a strong manuscript. I have just a handful of comments/queries.
1) For the readers not expert in proteomics, it would be important to explain why the authors searched for proteins as potential drug targets. Please, reference the seminal study by Santos et al, A comprehensive map of molecular drug targets. Nat Rev Drug Discov 16, 19–34 (2017).* [*https://doi.org/10.1038/nrd.2016.230.*](https://nam02.safelinks.protection.outlook.com/?url=https%3A%2F%2Furldefense.com%2Fv3%2F__https%3A%2Fdoi.org%2F10.1038%2Fnrd.2016.230.__%3B!!NLFGqXoFfo8MMQ!raG-g30ZJ01Sws2eCMnHc5nJdmwaaTDFbVDKCiCX7PB3NcisdKzGDd_TQ0LYbWMqNF7WVisk96w60FIWh8uhryD8MALawxrGIqg%24&data=05%7C02%7Cchen.li%40bms.com%7Ca224873368b545b6c27708dcafd3c59b%7C71e34cb83a564fd5a2594acadab6e4ac%7C0%7C0%7C638578571305570028%7CUnknown%7CTWFpbGZsb3d8eyJWIjoiMC4wLjAwMDAiLCJQIjoiV2luMzIiLCJBTiI6Ik1haWwiLCJXVCI6Mn0%3D%7C0%7C%7C%7C&sdata=z8YoP8Fmjb7J%2FlWda4EomdrCJliim0o0kuTedO51axw%3D&reserved=0) *Santos et al reviewed all the FDA approved drugs as of 2016 and found that 95% of all drugs targets turned out to be proteins. So drug discovery largely revolves around proteins. If the authors have the bandwidth, they could update the published numbers but if not, they should just reference the study.*

We thank the reviewer’s recognition of this work and invaluable comments. We added a sentence “…and 97% of therapeutic targets for FDA-approved drugs as of 2016 are proteins” in the introduction at line 25 and cited the Santos et al paper.

*2) Among the four MR methods used, some are suitable for a single pQTL while others require 2 or more pQTLs for optimal performance. If this reviewer understands it correctly about 59% of all proteins measured by Olink in UK BioBank had at least one pQTL but it is not clear how many had multiple (two or more) pQTLs. Please, present this information and discuss which MR methods work well with just a single pQTL and which require two or more pQTLs for reliable performance.*

Thank you for raising the question. **We assessed the number of proteins that have multiple pQTLs**. There are 188 and 220 proteins that have only one pQTL for analyses with cis- + trans-pQTLs combined or cis-pQTLs only, respectively, among which 5 proteins are significant for the former analysis and 1 for the latter. We used the Wald ratio MR method for proteins with only one pQTL. There are 2,483 proteins that have >1 pQTLs for the analysis with cis- + trans-pQTLs combined and 1,816 proteins for the analysis with cis-pQTLs, among which 188 and 113 are significant in the IVW method, respectively. We used IVW, MR Egger, weighted median and weighted mode methods for proteins with more than one pQTLs. In the final results we presented in the manuscript, we didn’t include results for proteins with only one pQTL (i.e. from Wald ratio analyses), because of its lack of reproducibility with other MR methods. **We removed the Wald ratio method and clarified this in the methods at line 69.**

*3) Unlike in the main text, Figure 1 shows that five different MR methods were used. Was it four (based on the text) or five (based on the Figure)?*

We thank the reviewer’s correction. We removed the Wald ratio method mentioned in Figure 1 because of the reasons illustrated in the repones to the previous comment, and modified the figure to include additional sensitivity analyses conducted.

*4) FinnGen cohort had relatively few participants – 1,225. How did that affect the power for MR validation?*

We thank the reviewer’s question. There was a total of 136 out of 227 proteins that were nominally significant in the FinnGen cohort (93 with cis- + trans-pQTLs and 84 with cis-pQTLs only), 108 reached FDRBH-adjusted P-value < 0.05 (92 with cis- + trans-pQTLs and 57 with cis-pQTLs only), and 57 proteins (56 with cis- + trans-pQTLs and 21 with cis-pQTLs only) reached Bonferroni corrected significance threshold. Most of the validated targets also showed consistent directionality of effects. There were 60.0% significant proteins replicated in the FinnGen cohort, and the unreplicated signals can be due to a lack of power. With the FinnGen Olink dataset, it is estimated to have 80% power to detect an odds ratio of 1.006 per 1-SD genetically predicted increase of circulating protein level using a nominal significance level. With the UK Biobank Olink data, because of the up to 40X larger sample size, the detectable odds ratio can be reduced to 1.0002, therefore much more powerful to detect even extremely small effect sizes.

1	Foley, C. N. *et al.* A fast and efficient colocalization algorithm for identifying shared genetic risk factors across multiple traits. *Nat Commun* **12**, 764 (2021). <https://doi.org:10.1038/s41467-020-20885-8>

2	Wallace, C. A more accurate method for colocalisation analysis allowing for multiple causal variants. *PLoS Genet* **17**, e1009440 (2021). <https://doi.org:10.1371/journal.pgen.1009440>

3	Theodoris, C. V. *et al.* Transfer learning enables predictions in network biology. *Nature* **618**, 616-624 (2023). <https://doi.org:10.1038/s41586-023-06139-9>

4	Sun, B. B. *et al.* Plasma proteomic associations with genetics and health in the UK Biobank. *Nature* (2023). <https://doi.org:10.1038/s41586-023-06592-6>

5	Verbanck, M., Chen, C. Y., Neale, B. & Do, R. Detection of widespread horizontal pleiotropy in causal relationships inferred from Mendelian randomization between complex traits and diseases. *Nat Genet* **50**, 693-698 (2018). <https://doi.org:10.1038/s41588-018-0099-7>

6	Zhu, Z. *et al.* Causal associations between risk factors and common diseases inferred from GWAS summary data. *Nat Commun* **9**, 224 (2018). <https://doi.org:10.1038/s41467-017-02317-2>

7	Lorincz-Comi, N., Yang, Y., Li, G. & Zhu, X. MRBEE: A bias-corrected multivariable Mendelian randomization method. *HGG Adv* **5**, 100290 (2024). <https://doi.org:10.1016/j.xhgg.2024.100290>

8	Zuber, V., Colijn, J. M., Klaver, C. & Burgess, S. Selecting likely causal risk factors from high-throughput experiments using multivariable Mendelian randomization. *Nat Commun* **11**, 29 (2020). <https://doi.org:10.1038/s41467-019-13870-3>

9	Chen, L. *et al.* Systematic Mendelian randomization using the human plasma proteome to discover potential therapeutic targets for stroke. *Nat Commun* **13**, 6143 (2022). <https://doi.org:10.1038/s41467-022-33675-1>

10	Shah, S. *et al.* Genome-wide association and Mendelian randomisation analysis provide insights into the pathogenesis of heart failure. *Nat Commun* **11**, 163 (2020). <https://doi.org:10.1038/s41467-019-13690-5>

11	Zheng, J. *et al.* Phenome-wide Mendelian randomization mapping the influence of the plasma proteome on complex diseases. *Nat Genet* **52**, 1122-1131 (2020). <https://doi.org:10.1038/s41588-020-0682-6>

12	Chaffin, M. *et al.* Single-nucleus profiling of human dilated and hypertrophic cardiomyopathy. *Nature* **608**, 174-180 (2022). <https://doi.org:10.1038/s41586-022-04817-8>

13	Liu, X. *et al.* Lineage-specific regulatory changes in hypertrophic cardiomyopathy unraveled by single-nucleus RNA-seq and spatial transcriptomics. *Cell Discov* **9**, 6 (2023). <https://doi.org:10.1038/s41421-022-00490-3>

14	Huang, G. *et al.* Titin-truncating variants in hiPSC cardiomyocytes induce pathogenic proteinopathy and sarcomere defects with preserved core contractile machinery. *Stem Cell Reports* **18**, 220-236 (2023). <https://doi.org:10.1016/j.stemcr.2022.11.008>

15	Sakaue, S. *et al.* A cross-population atlas of genetic associations for 220 human phenotypes. *Nat Genet* **53**, 1415-1424 (2021). <https://doi.org:10.1038/s41588-021-00931-x>

16	Tadros, R. *et al.* Shared genetic pathways contribute to risk of hypertrophic and dilated cardiomyopathies with opposite directions of effect. *Nat Genet* **53**, 128-134 (2021). <https://doi.org:10.1038/s41588-020-00762-2>

17	Minelli, C. *et al.* The use of two-sample methods for Mendelian randomization analyses on single large datasets. *Int J Epidemiol* **50**, 1651-1659 (2021). <https://doi.org:10.1093/ije/dyab084>

We thank the reviewer’s further comments for helping to improve the manuscript. Here are our revisions and responses to the comments.

*Thanks. The addition of a table specifying ICD-10 codes used is helpful. However, it should be noted that for UK Biobank these come from hospital episode statistics. Primary care data is also available for around 250,000 participants in UK Bioban, and it is known that the use of both sources of data (from primary and secondary care) can enhance capture of diseases such as MI. (Herrett E, Shah AD, Boggon R, Denaxas S, Smeeth L, van Staa T, Timmis A, Hemingway H. Completeness and diagnostic validity of recording acute myocardial infarction events in primary care, hospital care, disease registry, and national mortality records: cohort study. BMJ. 2013 May 20;346:f2350. doi: 10.1136/bmj.f2350. PMID: 23692896; PMCID: PMC3898411.) The authors should acknowledge the limitation of not including diagnostic information from primary care records in the analysis.*

We acknowledged the limitation in the method section.

*Thanks, but my comments still stand and I believe that these two designations should be removed from the analysis which is motivated by the search for new therapeutic targets. Cardiomegaly is a morphological description that can arise from a number of underlying conditions and would not form the subject of a drug development programme. Oesophageal varix is a complication of chronic liver disease not a cardiovascular disease. Simply because an ICD-10 code exists does not justify its use in this type of research without further critical assessment. To use some extreme examples to illustrate my point, ICD-10 codes exist for the following conditions: Walked into lamppost, subsequent encounter (W220.2XD); Pecked by a chicken. (W61.33); Struck by duck, subsequent encounter (W61.62XD) and Bizarre personal appearance (R46.1).*

We removed the cardiomegaly and oesophageal varix from the analyses.

*Thanks – the addition of the so-called pleiotropy-robust methods for MR analysis does not properly address my point. Those methods were designed for non-protein exposures such as lipids and blood pressure for which there is no alternative. Where proteins are the exposure of interest, the most pleiotropy robust approach is to restrict the genetic instruments to those in the vicinity of the gene encoding the protein of interest, i.e cis-MR and this should be the primary analysis in a paper of this type. The reasoning has been amply discussed in the references listed in my earlier comment. All the pleiotropy robust methods used carry their own assumptions and should not be seen as simply removing any effect of horizontal pleiotropy. It is concerning that despite the issue being raised here and by other reviewers that the MR analyses methods section still leads with detailed descriptions of genome wide methods with cis-MR being mentioned only as a sensitivity analysis, and not referenced (see list of references in my earlier comment) or described. This remain a serious flaw. The section in the Discussion in which genome wide and cis-MR are dealt with as equequal alternatives with a pro and con discussion (lines 615-620) is incorrect. The primary stated aim of the analyses done was to identify the causal relevance of proteins for CVDs for which cis-MR should be the primary analysis. The argument used that cis + trans-MR has ‘greater statistical power’ omits the clause ‘to produce a biased estimate’.*

We restructured the manuscript and rephrased our description in the methods and results to better highlight cis-MR as a primary analysis. We also added all references from the reviewer’s earlier comment.

*The definition of biomarker used in this response – ‘proteins whose plasma levels are causally influenced by CVD’ – is different from that in their original submission ‘biomarkers that vary protein expressions as consequences of diseases’. The authors should decide what their definition of a biomarker is. Moreover, the justification for ‘reverse MR’ is to identify proteins whose plasma levels are causally influenced by CVD, but I don’t see why this would be important. It is of interest to know if a disease affects the level of a protein because such a protein might be useful for early disease detection, but for this application causality is not the issue: one can simply directly observe the direct association of the disease and protein. In forward MR, where knowing if a protein exposure is a cause of disease is important because targeting it pharmacologically could help prevent or treat the disease. What is the translational application of reverse MR? Moreover, reverse MR is very substantially prone to horizontal pleiotropy because the instruments used to index the exposure (the disease) are selected from throughout the genome and so could have direct pathways to the biomarker independent of the disease. Moreover, reverse MR can lead to flawed inference. For example, genetic variants identified for an association with MI in a GWAS include those affecting blood LDL-C. A reverse MR of CHD on blood lipids might then lead to the erroneous inference that MI causes an elevation in LDL-C. I restate my view that the reverse MR analyses should be dropped. That previous publications have reported findings from ‘reverse-MR analysis is not a justification repeating the mistake here.*

We reclarified our definition for the biomarkers – proteins whose plasma levels are affected by the disease status (i.e. proteins serve as outcomes and diseases as exposures in the MR analysis). We further added limitations of the reverse MR in the discussion at line 529-535 as the reviewer pointed out. We also added an acknowledgement of the challenges in translating the findings from reverse MR into concrete clinical conclusions. We think there are still values in keeping the reverse MR analyses, and hope with the necessary acknowledgement of the limitations, the reviewer is more satisfied.

*The acknowledgments are incomplete and several of the points I raised appear to have been ignored. The acknowledgement lists the pharmaceutical company partners but does not list the public bodies, charities, and donors that funded the set-up and follow up of UK Biobank. I am again providing a link to the webpage which details the funders. https://www.ukbiobank.ac.uk/learn-more-about-uk-biobank/about-us/our-funding. The disease outcomes in UK Biobank come from linked electronic health record data and so acknowledgement should be given to the UK National Health Service which, provides health care, free at the point of delivery funded through general taxation. To re-re-iterate, given the authors are all employees and stockholders of BMS, can they provide written reassurance that all findings made as part of this analysis are disclosed in this publication (as would be expected in a research paper) and that none have been held back for commercial reasons. The following statement in the acknowledgement is ambiguous: ‘..providing early access to individual-level data to funding partners..’ Early access was not provided to all the funding partners of UK Biobank but exclusively to pharmaceutical company partners of UKB-PPP. That should be clarified.*

We added the public bodies, charities, and donors that funded the set-up and follow up of UK Biobank in the acknowledgement. We also added acknowledgement to the UK National Health Service. We added the disclosure that all findings made as part of this analysis are disclosed in this publication.

R1- Response to new reviewers: 18-Feb-2025

*Reviewer #1: The manuscript "Proteome-wide Mendelian Randomization Identifies Candidate Causal Proteins for Cardiovascular Diseases" presents a well-structured and robust study integrating genetics, proteomics, and single-cell transcriptomics to identify potential therapeutic targets for cardiovascular diseases. The study is commendable for its large-scale data integration, bidirectional Mendelian Randomization analysis, and multi-ancestry approach. While the manuscript is scientifically rigorous, several minor revisions could enhance clarity, interpretation, and presentation. Below are comments followed by specific questions for the authors. 1. The manuscript provides bidirectional MR analysis, but the distinction between causal proteins and biomarkers remains somewhat abstract. It would be helpful to include a clearer conceptual figure summarizing key findings (e.g., Venn diagram of shared vs. distinct causal proteins and biomarkers). The study addresses pleiotropic effects of proteins, particularly those involved in immune response and inflammation. However, further discussion on how pleiotropy impacts drug discovery and interpretation of MR findings would be beneficial.*

We appreciate the reviewer’s comments and add the Venn diagram to summarize the key findings in supplementary figure 1. We agree that understanding the pleiotropic effects of proteins is crucial for interpretation of the MR findings and has important implications for drug discovery, and we add a discussion paragraph (the third paragraph under the title “Pleiotropy in proteomic MR” in the discussion) talking about the pleiotropy at the protein level.

*2. How were confounders addressed in the MR analysis to mitigate horizontal pleiotropy, and how do the authors ensure robustness of causal inference? Given the bidirectional MR approach, do any of the identified proteins show evidence of both forward and reverse causality? If so, how should these be interpreted in a therapeutic context?*

We thank the reviewer’s valuable questions. We added a discussion paragraph (the second paragraph under the title “Pleiotropy in proteomic MR” in the discussion) talking about the horizontal pleiotropy at the variant (pQTL) level. Basically, we addressed the horizontal pleiotropy by 1) sensitivity analyses using MR-PRESSO and GSMR that filter out pleiotropic outliers, as well as a multivariable MR method – MRBEE that uses multiple proteins with shared genetic instruments -- pQTLs as exposures and estimates independent effects of individual proteins, and 2) validation analyses using independent proteomics data from FinnGen. We assessed the robustness of causal inference based on the replication in sensitivity analyses and independent cohort analyses, as well as orthogonal evidence from literature and single cell transcriptomic data. response letter to reviewers Yes, there are two proteins – MMP12 and LGALS4 that are significant in both forward and reverse MR. This suggested that the two proteins are likely to be both causes and consequences of the relevant diseases, but it is hard to know the dynamic changes of these two proteins along the disease trajectory. The protein levels may change before the disease incidences, therefore are prospectively associated with the disease risks – likely causal for the diseases, and remain abnormal after the disease occurrences, therefore reversely associated with the diseases – likely responsive for the diseases. The key question is to fully understand protein-level changes in different stages during disease development to distinguish disease incidence and progression, which is hard to address given the limitations on disease endpoint definitions for GWAS being mostly incidences. We further developed our discussion on MMP12 as an example in the second paragraph under the title “The values and limitations of bidirectionality in MR analyses” in the discussion.

*3. BTN3A2 was identified as a novel protective factor for ischemic stroke. What biological mechanisms might explain this effect, and how does it compare to existing stroke-related immune targets?*

We appreciate the reviewer’s valuable question about the BTN3A2 as a novel target for ischemic stroke. Based on the MR results and literature review, we proposed that higher BTN3A2 levels may decrease the risk of ischemic stroke by dampening detrimental immune reactions that may exacerbate ischemic injury. BTN3A2 belongs to the butyrophilin family, which is involved in immune modulation, particularly through the activation of Vγ9Vδ2 T cells. These cells are a subset of γδ T cells that play a protective role in inflammation by recognizing dangerassociated determinants up-regulated in immune responses to tumor cells and infections.1 Agonists of BTN3A2 can activate the Vγ9Vδ2 T cells that provide immune surveillance and reduce neuroinflammation and tissue damage in ischemic stroke.

A common therapeutic approach for stroke focuses on inhibiting pro-inflammatory cytokines, such as IL-6 or TNF-α, which are involved in the inflammatory response and neuroinflammation post-stroke. For example, IL-6 has been widely studied for its role in stroke, and therapies aimed at blocking IL-6 have shown promise in reducing infarct size and improving recovery of ischemic stroke patients in randomized controlled trials and systematic review of prospective studies. 2,3 Similarly, TNF-α inhibitors have been explored as potential stroke therapies due to their ability to reduce neuroinflammation and brain tissue damage.4 BTN3A2 shares some similarities with existing stroke-related immune targets, in which they are all involved in attenuating post-stroke inflammation, but BTN3A2 offers a distinct mechanism of action for immune-related therapeutic targets for stroke. Agonists of BTN3A2 provide a novel therapeutic target for resetting and restoring homeostasis of immune system through activation of the Vγ9Vδ2 T cells, rather than broadly suppressing immune responses seen with cytokine inhibitors, such as IL-6 and TNF-⍺. 5 The specialized subset of T cells -- Vγ9Vδ2 T cells function as sentinels to guard and repair inflammation-induced neuronal cell stress and damage that have occurred in ischemic stroke. In addition, BTN3A2’s potential to regulate immune responses through its interaction with other butyrophilins, such as BTN2A1, which is involved in pathogen recognition and immune activation, further supports the hypothesis that butyrophilinsmediated Vγ9Vδ2 T cell activation could modulate stroke-related immune mechanisms in a more precise way compared to traditional anti-inflammatory therapies. This may represent a novel therapeutic avenue for ischemic stroke that balances immune regulation and tissue protection rather than suppressing the immune system globally. **We summarized and added this part to the discussion under the title “Novel potential therapeutic targets for CVD”.**

4. *What are the most promising druggable targets identified in this study, and how do they compare to known cardiovascular drug targets? How do the authors envision integrating MR based target discovery with functional validation (e.g., CRISPR screening, knockout mouse models) to advance novel therapies for CVD?*

We than the reviewer’s inspiring comment. We identified potential targets for CVDs, particularly through modulating immune response, vascular remodeling and metabolic processes. Among these novel targets, BTN3A2 stands out as a particularly promising target for ischemic stroke, PAM for stable angina pectoris and LPL for peripheral artery disease. The comparison of BTN3A2 to known ischemic stroke targets has been elucidated in the response to the last comment. Such immune-based mechanism also offers an alternative therapeutic strategy to more conventional lipid-targeting approaches, such as PCSK9 for lowering LDL cholesterol, or vascular remodeling mechanisms, such as ECE1 for hypertension and heart failure. PAM is a protein predominantly expressed in cardiomyocytes, where it plays a crucial role in the biosynthesis of proANP, a precursor to the biologically active ANP. ANP is a potent vasodilator that helps modulate blood pressure and fluid retention, which are crucial in heart failure or other related cardiac intrinsic CVD management. Unlike other heart failure therapies, such as ACE inhibitors and angiotensin receptor blockers, whose target genes are not expressed in cardiac muscles, PAM expression is highly enriched in cardiomyocytes and offers a unique mechanism of action to treat heart failure through regulating ANP biosynthesis. LPL has a well-established role in lipid metabolism, but its cell-specific effects in cardiomyocytes link it directly to cardiac pathophysiology. Comparing to known targets like PCSK9, which is dominantly expressed in the liver, LPL may exert effects in the cardiomyocytes and adipocytes, providing a distinct opportunity to design drug modalities to specifically target these two cell types for CVD indications. To advance these novel therapies, integrating the MR findings with functional validation is essential. The next step in validating these targets involves using approaches such as CRISPR-based gene editing and genetically modified mouse models to test the biological effects of these proteins in vitro and in vivo. **We emphasized the importance for functional validation under the title “Novel potential therapeutic targets for CVD”.**

In conclusion, this manuscript provides a valuable contribution to cardiovascular proteomics and drug discovery.

*Reviewer #2: The reviewer would like to acknowledge that they were not involved in previous rounds of review and recruited during the latest round of revision. I have reviewed both R2 and R3 versions of the manuscript. The study presented in the manuscript evaluates causal relationships between 2,940 circulating proteins and 19 cardiovascular diseases (CVD) using biobank-scale genetic association summary statistics within a bi-directional Mendelian randomization (MR) framework. It consists of two main components: primary analyses to disentangle causal relationships and secondary analyses aimed at strengthening evidence for target prioritization. The study highlights the identification of novel candidate causal signals for CVD and suggests these findings can contribute to drug discovery efforts. Overall, this approach is impactful in helping driving future drug discovery for CVD, making it highly relevant to a broad audience of this journal. In their response to prior reviewer comments, the authors articulated their rationale for incorporating both reverse MR and trans-pQTL analyses, which I found to be well-founded and adept. Therefore, I concur with previous reviewers that the section on reverse MR should be retained for its potential to inspire future biomarker discovery.*

We very much appreciate the reviewer’s positive feedback on our work.

1. Bonneville M, O'Brien RL, Born WK. Gammadelta T cell effector functions: a blend of innate programming and acquired plasticity. Nat Rev Immunol. 2010;10:467-478. doi: 10.1038/nri2781

2. Papadopoulos A, Palaiopanos K, Bjorkbacka H, Peters A, de Lemos JA, Seshadri S, Dichgans M, Georgakis MK. Circulating Interleukin-6 Levels and Incident Ischemic Stroke: A Systematic Review and Meta-analysis of Prospective Studies. Neurology. 2022;98:e1002-e1012. doi: 10.1212/WNL.0000000000013274

3. Chu X, Ma Z, Liu Y, Sun J, Wang N, Li C, Feng X, Li J, Wang B, Zhou C, et al. IRIS, a randomised, double-blind, placebo-controlled trial of interleukin-6 receptor inhibition undergoing endovascular treatment in acute anterior circulation ischaemic stroke: study rationale and design. Stroke Vasc Neurol. 2024. doi: 10.1136/svn-2024-003574

4. Joseph AM, Karas M, Jara Silva CE, Leyva M, Salam A, Sinha M, Asfaw YA, Fonseca A, Cordova S, Reyes M, et al. The Potential Role of Etanercept in the Management of Post stroke Pain: A Literature Review. Cureus. 2023;15:e36185. doi: 10.7759/cureus.36185

5. Ramirez-Valle F, Maranville JC, Roy S, Plenge RM. Sequential immunotherapy: towards cures for autoimmunity. Nat Rev Drug Discov. 2024;23:501-524. doi: 10.1038/s41573- 024-00959-8
